# Nutritional Metabolites of Red Pigmented Lettuce (*Lactuca sativa*) Germplasm and Correlations with Selected Phenotypic Characters

**DOI:** 10.3390/foods10102504

**Published:** 2021-10-19

**Authors:** Awraris Derbie Assefa, On-Sook Hur, Bum-Soo Hahn, Bichsaem Kim, Na-Young Ro, Ju-Hee Rhee

**Affiliations:** National Agrobiodiversity Center, National Institute of Agricultural Sciences, RDA, Jeonju 54874, Korea; awraris@korea.kr (A.D.A.); oshur09@korea.kr (O.-S.H.); bshahn@korea.kr (B.-S.H.); bsam92@korea.kr (B.K.); nonanona@korea.kr (N.-Y.R.)

**Keywords:** *Lactuca sativa*, UPLC-DAD-QTOF/MS, phenolic compounds, anthocyanins, hydroxycinnamoyl derivatives, flavones, flavonols, PCA, PLS-DA, phenotypic characters

## Abstract

Lettuce is an important dietary source of bioactive phytochemicals. Screening and identification of the health beneficial metabolites and evaluating the relationships with phenotypic characters can help consumers adjust their preferences for lettuce plant types. Thus, we explored the major health-beneficial individual metabolites and antioxidant potential of 113 red pigmented lettuce leaf samples. A UV–Vis spectrophotometer and UPLC-DAD-QTOF/MS (TQ/MS) instruments were used for the identification and quantification of metabolites and antioxidant activity accordingly. The metabolites were quantified against their corresponding external standards. The contents of metabolites varied significantly among lettuce samples. Cyanidin 3-*O*-(6″-*O*-malonyl)glucoside (4.7~5013.6 μg/g DW), 2,3-di-*O*-caffeoyltartaric acid (337.1~19,957.2 μg/g DW), and quercetin 3-*O*-(6″-*O*-malonyl)glucoside (45.4~31,121.0 μg/g DW) were the most dominant in red pigmented lettuce samples among anthocyanins, hydroxycinnamoyl derivatives, and flavonols, respectively. Lettuces with dark and very dark red pigmented leaves, circular leaf shape, a strong degree of leaf undulation, and highly dense leaf incisions were found to have high levels of flavonoids and hydroxycinnamoyl derivatives. Principal component analysis was used to investigate similarities and/or differences between samples, and the partial least square discriminant analysis classified them into known groups. The key variables that contributed highly were determined. Our report provides critical data on the bioactive constituents of red pigmented lettuce to breeders developing varieties with enhanced bioactive compounds and to nutraceutical companies developing nutrient dense foods and pharmaceutical formulations.

## 1. Introduction

Lettuce (*Lactuca sativa* L., Family Asteraceae) is among the most popular vegetables, is consumed either fresh or in salad mixes, and is highly ranked in production and economic value [1]. Planted annually in backyards, shade nets, containers, or greenhouses in a range of environmental conditions, lettuce also provides important economic benefits due to suitability to vertical farming systems [2]. Consumers’ interest in lettuce is increasing because of its superior visual quality, low calorie make-up, minimal microbial load, and high contents of beneficial phytochemicals [1]. According to the Food and Agriculture Organization of the United Nations’ (FAO, 2019) report [3], worldwide lettuce and chicory production has increased from 11 million tons in 2000 to more than 29 million tons in 2019, from which ~56.0% was produced by China, followed by the USA (~12%) and India (~4.3%). Cultivated lettuce groups are of various types, including cos (romaine), leafy (cutting), stalk (asparagus), butterhead, crisphead (iceberg or cabbage), Latin, and oilseed. The growth habit, leaf texture, shape, and color vary among groups [4]. 

The nutraceutical property of lettuce varies based on various factors. Reports indicate the romaine and leafy types of lettuce have substantial amounts of ascorbic acid, vitamin A, carotenoids, and folate, whereas the crisphead contains relatively low amounts of these compounds [4]. Significant variations in primary and secondary metabolites were observed between the head and leaf lettuce types [5]. In addition to growth type, genetic variation also affects the nutritional and phytochemical properties of lettuce [6]. Nutritional contents such as protein, sugars, lipids, minerals, vitamins, and carotenoids of romaine, butterhead, cutting, crisphead, and stalk lettuce types were compiled in a previous report [4]. Other naturally available primary and secondary metabolites with benefits to human health, such as phenolic acids, flavonoids [2,7,8], tocopherols, carotenoids [6], and sesquiterpene lactones [5] were also reported earlier.

Anthocyanins are a naturally occurring family of flavonoids responsible for some of the red, purple, orange, and blue pigmentation of fruits and vegetables [9]. Red pigmented lettuce accumulates a large amount of anthocyanins. Anthocyanins are generally present in the form of anthocyanidin glycosides and acylated anthocyanins [10]. Anthocyanins’ pigments extracted from plants have been traditionally used as dyes, food colorants, and as medicines to treat various diseases [9,10]. A great deal of epidemiological evidence exists on the involvement of anthocyanins in the prevention of cardiovascular disease [9]. Several reports also showed that anthocyanins exhibit antioxidant [11], antidiabetic [12], anticancer [13], anti-inflammatory [14], antimicrobial [15], antitumor [16], antimutagenic [17], and antiobesity [18] effects.

The hydroxycinnamic acids are the most abundant metabolites in lettuce, followed by the flavonoids [8]. Hydroxycinnamic acids have widely recognized uses, such as cosmeceutical applications [19], inhibition of human low-density lipoprotein oxidation [20], antioxidant effects [21,22], and other health-related benefits (improvements in blood pressure, metabolic pressure, and certain cancers [21,23], among others). The chemistry, biosynthesis, and bioactivity of caffeoylquinic acids, derivatives of hydroxycinnamic acids, were recently reviewed [23]. A wide range of flavonoids, characterized by variable phenolic structures, have been reported to be in lettuce [8,24]. Flavonoids are considered an indispensable component in various pharmaceutical, medicinal, cosmetic, and nutraceutical applications [25] and possess antioxidant activities [26]. 

Quantitative data on major health beneficial metabolites of red pigmented lettuce using a large population of samples are illusive. In this study, with the help of the UPLC-DAD-QTOF/MS (TQ/MS) instruments and UV–Vis spectrometry, we identified and quantified three anthocyanins, four hydroxycinnamoyl derivatives, two flavonols, and one flavone in 113 samples of germplasm and commercial cultivars of lettuce at the mature stage. Additionally, we have assessed the total phenolic contents (TPC) and 2,2′-azino-bis-(3-ethylbenzothiazoline-6-sulphonic acid) (ABTS) radical scavenging potentials. The relationships between biochemical and phenotypic traits were also investigated. Our results provide critical data for lettuce breeders developing new varieties with enhanced levels of bioactive compounds for nutraceutical applications.

## 2. Materials and Methods

### 2.1. Reagents, Standards, and Chemicals

All chemicals and reagents used in the extraction and analysis, unless specified, were of analytical grade and purchased from Fisher Scientific Korea Ltd. (Seoul, Korea) and Sigma-Aldrich (St. Louis, MO, USA). The standard of cyanidin 3-*O*-glucopyranoside (C3-G) was purchased from Extrasynthese (Lyon, France). Standards of gallic acid, TROLOX (6-hydroxy-2,5,7,8-tetramethylchromane-2-carboxylic), 3-*O*-caffeoylquinic acid (3-CQA), 5-*O*-caffeoylquinic acid (5-CQA), 2,3-di-*O*-caffeoyltartaric acid (2,3-DCTA), 3,5-di-*O*-caffeoylquinic acid (3,5-DCQA), quercetin 3-*O*-glucuronide (Q3-G), quercetin 3-*O*-(6″-*O*-malonyl)glucoside (Q3-6″MG), and luteolin 7-*O*-glucuronide (L7-G) were purchased from Sigma-Aldrich (St. Louis, MO, USA). 

### 2.2. Plant Materials

**Figure 1:**
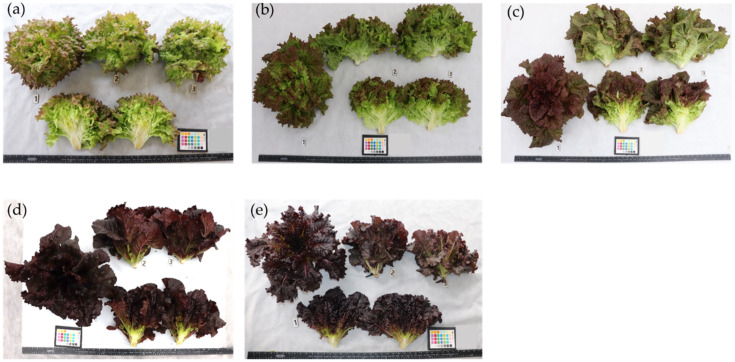
Representative photos of lettuce samples with various intensities of red pigmentation. Very light (**a**), light (**b**), medium (**c**), dark (**d**), and very dark (**e**).

Lettuce samples were grown at a research field of National Agrobiodiversity Center (NAC), Rural Development Administration (RDA), Jeonju (35°49′18″ N, 127°08′56″ E), Republic of Korea. Seeds of 105 germplasms and 8 commercial cultivars obtained from the gene bank of NAC were sown in plug trays, and seedlings were grown in a greenhouse. RDA’s recommended agricultural management practices for lettuce were followed. Briefly, four week old seedlings were transplanted to the field of a plastic house with a planting density of 20 × 20 cm. Each sample consisted of 24 plants. Mature lettuce leaves, defined by the head/leaf reaching a marketable size, were harvested between 65 and 75 days after sowing. Harvested samples were immediately transported to the laboratory, placed in vinyl freezer bags, and held at −80 °C until further treatment. The frozen samples were subsequently lyophilized for 48 h using a vacuum freeze-drier (Ilshibiobase, Rijssen, Netherlands). Freeze-dried samples were ground to a fine powder using a mortar and pestle, and stored at −20 °C for the subsequent analysis. The experimental design was completely randomized and incorporated biological triplicates. Representative photos of lettuce samples with various intensities of red pigmentation are presented in Figure 1. 

### 2.3. Description of the Morphological Characters of Lettuce Samples

Morphological characters were recorded based on physical observations in the field and in the laboratory and are presented in Table S1. The morphological characters were evaluated based on modified descriptors of the International Union for the Protection of New Varieties of Plants (UPOV) for lettuce [27]. Additional pictorial explanation of selected phenotypic characters is presented in a Supplementary File (Figure S2). The leaf length, width, and the weight of lettuce plant were evaluated with the help of a meter and digital balance accordingly. Other qualitative morphological characters, such as color of the cotyledon, plant growth type, leaf shape, leaf attitude, leaf blade (degree of undulation of the margin and density of incisions on the margin on the apical part), and intensity of red color of the outer leaves were also examined. 

### 2.4. Extraction and Analysis of Phenolic Compounds and Antioxidants

#### 2.4.1. Extraction

Extracts were prepared by homogenizing 25 mg of finely ground freeze-dried lettuce samples with 1 mL of acidified (1%, formic acid) methanol/water (5:1, *v*/*v*) mixture under a sonication bath at room temperature for 30 min. Then, extracts were centrifuged at 10,000× *g* for 10 min at 4 °C, and the supernatants were recovered. The residues were further extracted following the same step described above. The obtained supernatants were combined, filtered through a 0.25 μm polytetrafluoroethylene (PTFE) filter (Millipore Ltd., Bedford, MA, USA), dissolved in an appropriate concentration, and used for subsequent analysis.

#### 2.4.2. Determination of Total Phenolic Content (TPC) 

The Folin–Ciocalteu method [28] was used to determine the total phenolic content, as described earlier [29], with slight modifications. Briefly, to 100 μL of appropriately diluted sample solution, 100 μL Folin–Ciocalteu reagent was added and allowed to react at room temperature for 3 min. To this mixture, 100 μL solution of 2% sodium carbonate was added and incubated for 30 min. Blank was prepared using 80% aqueous MeOH in place of sample solution. Test solutions were prepared using a 96-well plate. Absorbance was measured at 750 nm using an Eon Microplate Spectrophotometer (Bio-Tek, Winooski, VT, USA). The external standard calibration curve was prepared using gallic acid, following a similar procedure to that described above. Results were calculated based on the calibration equation (Y = 5.184X + 0.0025, where Y stands for absorbance; X stands for concentration; R^2^ = 0.9998) derived from the calibration curve, and are expressed as μg of gallic acid equivalent per gram (μg GAE/g) of dry weight (DW) sample. 

#### 2.4.3. 2,2′-Azinobis-(3-ethylbenzothiazoline-6-sulfonic acid) (ABTS) Radical Scavenging Activity

ABTS radical scavenging activity was estimated using an improved ABTS decolorization assay by Re et al. (1999) [30] with slight modifications. Briefly, an ABTS radical cation was generated by reacting 7 mM ABTS with 2.45 mM potassium persulphate, followed by overnight incubation of the mixture in the dark at room temperature. The solution was further diluted with water until the absorbance reading at 734 nm equals 0.7 ± 0.02 and used for preparing the test solution. Test solutions (A_sample_, A_sample blank_, A_control_, and A_control blank_) were prepared using a 96-well plate. Then, 10 μL of appropriately diluted sample solution was mixed with 190 μL of ABTS radical cation solution and allowed to react for 30 min at room temperature fully covered with aluminum foil. Absorbance was determined using an Eon Microplate Spectrophotometer (Bio-Tek, Winooski, VT, USA) at 734 nm. The capability of lettuce leaf extract to scavenge the ABTS radical was calculated using the following Equation (1):

(1) $$\mathrm{ABTS}\;\mathrm{radical}\;\mathrm{scavenging}\;\mathrm{activity}=1-\frac{A_{\mathrm{sample}}-A_{\mathrm{sample}\;\mathrm{blank}}}{A_{\mathrm{control}}-A_{\mathrm{control}\;\mathrm{blank}}}$$

where A_sample_ = absorbance of mixture of ABTS cation solution (190 μL) and sample (10 μL); A_sample blank_ = absorbance of mixture of sample (10 μL) and water (190 μL); A_control_ = absorbance of mixture of ABTS cation solution (190 μL) and 80% aqueous MeOH (10 μL); and A_control blank_ = absorbance of mixture of water (190 μL) and 80% aqueous MeOH (10 μL). The radical scavenging activity was reported as μg TROLOX equivalent per gram (μg TE/g) DW obtained by comparing the results with a TROLOX calibration curve constructed following a similar experimental procedure described above. The calibration equation used for quantification was Y = 6.4879X + 0.0024 (R^2^ = 0.9983).

#### 2.4.4. Analysis of Hydroxycinnamoyl Derivatives and Flavonoids

Sample test solutions, extracted as described in Section 2.4.1, were subjected to an Agilent UPLC 1290 infinity system equipped with a photodiode array (PDA) detector (Agilent Technologies, Santa Clara, CA, USA). Chromatographic separation was performed on CORTECS^TM^ UPLC^®^ T3 C_18_ (1.6 μm id, 2.1 × 150 mm) column (Waters Co., Milford, MA, USA). The column thermostat was maintained at 30 °C. The solvent system consisted of 0.5% formic acid in water (mobile phase A) and 0.5% formic acid in acetonitrile (mobile phase B). The elution was started with a linear gradient of 5 to 25% of B for the first 20 min, followed by 25 to 50% of B for the last 5 min, and held to a post run time of 5 min to maintain its initial condition. The sample injection volume and mobile phase flow rate were kept at 2 µL and 0.3 mL/min, respectively. The signal acquisition wavelengths used were 320 and 350 nm for hydroxycinnamoyl derivatives and flavonoids, respectively. The quantification of compounds was performed using calibration equations (Figure S1) constructed using the corresponding commercial standards. Mass spectrometry was performed via a quadrupole time-of-flight (QTOF) mass spectrometry (Xevo G2-S, Waters MS Technologies, Manchester, UK) in electrospray ionization positive (ESI+) ion mode. The ESI-QTOF/MS instrument was operated based on the following settings: source and desolvation temperatures were 120 and 500 °C, respectively; capillary voltage, 3500 V; sample cone voltage, 40 V; desolvation gas, 1020 L/h; and cone gas, 50 L/h. Data were recorded in the mass range of 50–1000 *m*/*z* full scan mode.

### 2.5. Extraction and Analysis of Anthocyanins 

The extraction procedure of anthocyanin was adopted from a previous report [31], with slight modifications. Briefly, 50 mg of lyophilized lettuce samples were put into a 2 mL Eppendorf tube and extracted with 1 mL of methanol/water/acetic acid (85:15:0.5; *v*/*v*, MeOH/H_2_O/AcOH). The samples were vortexed for 1 min followed by sonication for 10 min. During sonication, the tube was shaken twice to re-suspend the sample. The solutions were kept at room temperature for 10 min, followed by centrifugation at 10,000× *g* for 10 min, and the supernatants were collected. The residues were re-extracted with 1 mL of the same solvent using a similar procedure described above. The supernatants of each sample were combined, filtered using a 0.45-μm polytetrafluoroethylene (PTFE) filter (Millipore Ltd., Bedford, MA, USA), and used for subsequent analysis. UPLC analysis was performed immediately after extraction. 

Separation of anthocyanins was conducted on Acquity UPLC BEH C_18_ column (2.1 mm × 150 mm, 1.7 μm) using Agilent UPLC 1290 infinity system equipped with photodiode array (PDA) detector (Agilent Technologies, Santa Clara, CA, USA). The column thermostat was maintained at 25 °C. The solvent system consisted of 1% formic acid in water (mobile phase A) and 1% formic acid in acetonitrile (mobile phase B). Isocratic elution was held at 10% B for the first min, followed by a linear gradient of 10–15% B from 1 to 5 min, 15–18% B from 5 to 12 min, and 18–90% B from 12 to 14 min; then an isocratic elution at 90% of B for a min; then a linear gradient of 90–10% B for another min; and finally, the initial conditions were held for the last four mins. The sample injection volume and mobile phase flow rate were kept at 3 µL and 0.25 mL/min, respectively. The signal acquisition wavelength was set to 520 nm. Mass spectrometry was performed on 6410 Triple Quad LC/MS (Agilent Technologies, Santa Clara, CA, USA) in precursor ion scanning mode equipped with Agilent MassHunter Workstation Data Acquisition software. For precursor ion scan, the instrument was tuned to the maximum abundance of the daughter ion (*m*/*z* 287). The quadrupole instrument was operated based on the following settings: capillary voltage, 3000 V; cone voltage, 35 V; desolvation gas temperature, 300 °C at a flow of 11 L/min, nebulizer pressure, 15 psi. The quantification of anthocyanins was done using a calibration equation (Y = 51354X + 36.237, R^2^ = 0.999; Y stands for peak area and X for concentration) constructed using the commercially available standard cyanidin 3-*O*-glucoside standard. Cyanidin 3-*O*-(3″-*O*-malonyl)glucoside and cyanidin 3-*O*-(6″-*O*-malonyl)glucoside were expressed as cyanidin 3-*O*-glucoside equivalents. To determine the specific quantities of cyanidin 3-*O*-(3″-*O*-malonyl)glucoside and cyanidin 3-*O*-(6″-*O*-malonyl)glucoside, the amounts calculated as cyanidin 3-*O*-glucoside equivalents were further multiplied by a molecular-weight correction factor. 

### 2.6. Statistical Analysis

Experiments were conducted in triplicates and biochemical data were recorded as mean ± standard deviation. Individual metabolites, TPC, and antioxidant levels were determined using regression equations of the calibration curves constructed using serial dilution experiments of external standards, gallic acid, TROLOX corresponding to the biochemicals accordingly. Duncan’s significant difference test (*p* < 0.05) performed by SPSS V25 statistical program (SPSS Inc., Chicago, IL, USA) was used for multiple comparisons. Pearson’s correlation analysis was also performed using the SPSS V25 statistical program. The PCA and PLS-DA were performed using SIMCA v13.0.3 software (Umetrics, Umea, Sweden).

## 3. Results and Discussions

### 3.1. Phenotypic Characters

**Figure 2:**
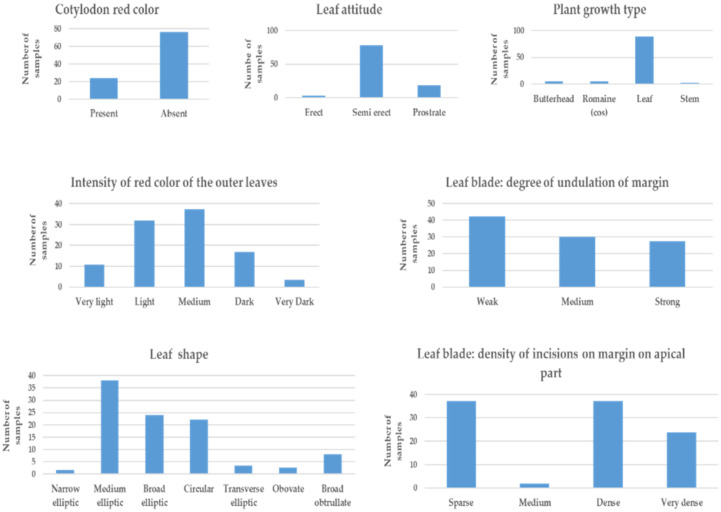
Frequency distribution of qualitative morphological characters of lettuce (*Lactuca sativa*) evaluated upon harvest maturity based on guidelines for the conduct of tests for distinctness, uniformity, and stability of modified International Union for the Protection of New Varieties of Plants (UPOV) descriptors.

The qualitative phenotypic characters of lettuce were described based on guidelines for the conduct of tests for distinctness, uniformity, and stability of modified International Union for the Protection of New Varieties of Plants (UPOV) [27] (Figure 2 and Table S1). About 76% of the samples contained red color at their cotyledon stage, and red color was absent in the remaining lettuce samples. While most of the analyzed samples were of leafy type (89.4%), there were also butterhead, romaine, and stem types, all combined representing 10.6% of the total samples. The majority of the sample (~80%) had semi-erect leaf latitude, and the remainder included erect and prostrate types. The shape of the lettuce leaf had a wide range of characteristics, from narrow elliptic to broad obtrullate. Medium elliptic, broad elliptic, and circular each represented 38.1, 23.9, and 22.1% of the total samples, respectively. The density of incisions of leaf margin was observed at the apical part on a scale of 1 to 4 (sparse, medium, dense, and very dense, respectively): sparse in 37.2%, dense in 37.2%, and very dense in 23.9% of the samples. The degree of undulation, evaluated on a scale of 1 to 3 (weak, medium, and strong, respectively) at the apical part, showed a weak degree of undulation in 42.5% of the samples; and 30.1% and 27.4% of the samples had medium and strong degrees of undulation. The other important character was the intensity of the red color of the outer leaves. The intensity of red color was evaluated on the outer leaf and graded from 1 to 5 (very light, light, medium, dark, and very dark, respectively). Most of the samples had medium and light intensity representing 37.2% and 31.9% of the total resources. The remaining 16.8%, 10.6%, and 3.5% of the samples had dark, very light, and very dark intensity of red color, respectively. The leaf length, leaf width, and leaf weight showed a wide range of variability among resources ranging from 13.5 to 97.1 cm, 8.7 to 28.2 cm, and 65.0 to 641.7 g, respectively.

### 3.2. Total Phenolic Content Antioxidant Activity

The total phenolic content (TPC) and antioxidant potential were quantified by UV–Vis spectrophotometry and are presented in a Supplementary File (Table S2). Significant variations in TPC and ABTS radical reducing potentials were observed among the genetic resources. The TPC and ABTS values ranged from 20,642.5 (IT No 100511, S/No 43) to 105,062.8 (IT NO 228749, S/No 86) μg GAE/g DW and 12,656.8 (IT No 100511, S/No 43) to 81,310.3 (IT NO 301289, S/No 102) μg TE/g DW. Fairly comparable amounts (TPC, 8600 to 63,800 μg GAE/g DW; ABTS, 6800 to 56,400 μg AAE/g DW) to our study were reported from baby leaf lettuce cultivars [1]. Llorach et al. (2008) [32] reported TPC content between 18.2 and 571.2 mg/100 g FW and ABTS antioxidant potential between 61.3 and 647.8 mg TEAC/100 g FW for five lettuce varieties and escarole. In another report, the TPC contents evaluated in five lettuce cultivars ranged between 13,900 and 46,900 μg GAE/g DW; the red leaf cultivars exhibited the highest amount [33]. Various factors, including genotype, variety, growth stage, and other cultural conditions affect the total phenolic content and antioxidant potentials of lettuce [32,33,34,35,36]. The phenotypic properties of lettuce showed significant differences in the antioxidant capacity and TPC (Table 1). For example, the mean TPC and antioxidant potential value of light + very light red-pigmented samples were significantly lower than medium and dark + very dark samples. Broad elliptic and circular leaf-shaped accessions were found to be superior to medium elliptic and broad obtrullate leaf shapes. The average TPC and ABTS values were also significantly higher in samples with strong leaf undulation compared to the medium and weakly undulated leaf margin samples (Table 1). The high levels of antioxidants and total polyphenols of lettuce samples make them food with high nutritional value. Hence, based on the evidence from our study, consumption of red pigmented, broad elliptic and circular leaf-shaped, and strong leaf undulated samples could help boost our immunity and minimize the onset of oxidative stress related diseases.

### 3.3. Individual Phenolic Compounds

#### 3.3.1. Anthocyanins

The UPLC chromatography of lettuce extract at 520 nm resulted in three detectable peaks at the retention times (t_R_) 5.93, 8.29, and 9.60 min (Figure S3). The t_R_ and the UV absorption maximum of the first peak (t_R_ 5.93) matched those of commercial standard cyanidin-3-*O*-glucoside. MS screening of the precursors of *m*/*z* 287 (cyanidin) detected two ions at *m*/*z* 449 and *m*/*z* 535. The first peak showed a molecular ion at *m*/*z* 449, which corresponds to the molecular cation of cyanidin 3-*O*-glucoside. Retention time, UV–Vis absorption spectra, and mass spectrometric fragmentation patterns of peak 1 were similar to the authentic standard cyanidin 3-*O*-glucoside, and hence, confirmed cyanidin 3-*O*-glucoside. Peaks 2 (t_R_ 8.29) and 3 (t_R_ 9.60) both had absorption maxima at 520 nm, a precursor ion at *m*/*z* 287, and a molecular ion at 535, indicating they could be malonilated cyanidin glucoside isomers. Based on a comparison of UV–Vis spectra, fragmentation patterns, and the elution order for cyanidin-glucosides on C_18_ column to previous reports on lettuce, peak 2 was tentatively identified as cyanidin 3-*O*-(3″-*O*-malonyl)glucoside and peak 3 as cyanidin 3-*O*-(6″-*O*-malonyl)glucoside [7,8,37,38,39]. Our previous report also indicated that acid hydrolysis of the anthocyanin extract of lettuce resulted in only cyanidin aglycone, confirming that all the anthocyanins identified in our study belong to conjugates of cyanidin [2]. Analysis was performed immediately after extraction to protect a possible conversion of compound cyanidin 3-(6″-malonyl)glucoside to cyanidin 3-(6″-malonyl)glucoside methyl ester and then to cyanidin 3-glucoside due to methylation or loss of the malonic acid moiety as previously reported [40]. 

Wide variation of anthocyanin concentration was observed among samples. Cyanidin 3-*O*-(6″-*O*-malonyl)glucoside was the most abundant anthocyanin (4.7 to 5013.6 μg/g DW) in all samples, contributing from 76 to 100% to the total anthocyanin content. Similar observations were reported previously from red lettuce [39,41], red onion [37,39], chicory, and endive [42]. Both cyanidin 3-*O*-glucoside and cyanidin 3-*O*-(3″-*O*-malonyl)glucoside were detected in quantifiable amounts in about 33% of the total samples, ranging from 14.2 to 422.9 μg/g DW and 4.6 to 201.3 μg/g DW, respectively. The concentrations of anthocyanins in all samples are presented in a Supplementary File (Table S2).

The significance of the effect of the intensity of red pigment on the concentration of anthocyanins was evaluated using Duncan’s test (Table 1). The dark + very dark red pigmented samples showed total anthocyanin concentrations of about 4.0 and 1.9 times higher than the light + very light and medium pigmented samples, respectively. The red pigment was highly associated with the content of anthocyanins, and this study is in agreement with previous findings [43,44]. Remarkable variability in anthocyanin concentrations was determined with leaf shape. The average anthocyanin concentrations of broad elliptic and circular samples were significantly higher than that of the broad obtrullate leaf-shaped samples. On the other hand, samples with strong degrees of undulation on the leaf margin appeared to be superior over samples with weak and medium degrees of undulation. While the effects of various factors such as storage temperature [45,46], leaf color [43,44], planting date, genotypes [5,43], postharvest processing [41], and cultivation methods [47,48] on the levels of lettuce anthocyanins were studied very well previously, the effects of some plant phenotypic characters, such as leaf shape and leaf blade, were ignored. As described in this study, these characters affect the levels of anthocyanins in lettuce, and the choice of plant phenotypic characters is also an important criterion to determine the nutritive index and health benefits of lettuce plants. 

Cyanidin 3-*O*-(6-*O*-malonyl)glucoside, residing exclusively in adaxial epidermal cells of leaves of lettuce, apart from its health related uses for humans, offers various benefits to the plant itself, such as removing oxygen radicals generated by chloroplasts, attenuating light, and offering effective and versatile protection to leaves without significantly compromising photosynthesis [49]. The high red pigmentation of the outer leaves of lettuce compared to the inner leaves observed in our samples could be related to the accumulation of high concentrations of cyanidin 3-*O*-(6-*O*-malonyl)glucoside. In concordance with our lettuce sample red coloration, the anthocyanins, which are directly related to the intensity of red pigment, were more abundant in older leaves *Quintinia serrata* [50]. It also exhibited antioxidant properties, partly related to prevention of DNA damage and intracellular reactive oxygen species (ROS) production, and anti-inflammatory activities in macrophages exposed to H_2_O_2_ and lipopolysaccharide through suppression of inflammatory mediators and protein expressions [51]. In addition, anthocyanins are also important food bioactive compounds from a technological point of view (improving the sensorial properties of food products and in food coloring). 

#### 3.3.2. Hydroxycinnamoyl Derivatives

Hydroxycinnamoyl derivatives were identified by comparison to the UV–Vis spectra, retention time, and MS fragment ions of the authentic analytical standards. The UPLC-PDA chromatogram of standard and lettuce extract is presented in Figure S4. Reversed-phase liquid chromatography using gradient acidic water and acetonitrile mobile phase and the C_18_ column used provided sufficient resolution. The retention times and UV absorption maxima of peaks 1, 2, 3, and 7 were matched that of authentic standards of 3-*O*-caffeoylquinic acid (3-CQA), 5-*O*-caffeoylquinic acid (5-CQA), 2,3-di-*O*-caffeoyltartaric acid (2,3-DCTA), and 3,5-di-*O*-caffeoylquinic acid (3,5-DCQA), respectively. The total ion current (TIC) extract of peaks 1 and 2 presented [M + H]^+^ ion at *m*/*z* 355 and fragment ions at *m*/*z* 181, 163, 145, and 135 indicating [caffeic acid + H]^+^, [caffeic acid + H − H_2_O]^+^, [caffeic acid + H − 2H_2_O]^+^, [caffeic acid + H − H_2_O − CO]^+^ respectively. The relative abundance was 100% for *m*/*z* 163 indicating losing the hydroxycinnamoyl moiety to be the easiest route of fragmentation as recently suggested [52]. The sodium adducts ([M + Na]^+^) and potassium ([M + K]^+^) were also observed at *m*/*z* 377 and 393, respectively. Hence, peaks 1 and 2 were identified as 3-CQA and 5-CQA. The presence of 2,3-di-*O*-caffeoyltartaric acid (Peak 3) was also further confirmed using its mass fragment ions. Although its [M + H]^+^ did not appeared in the mass spectra, the [M + Na]^+^ at *m*/*z* 497, and the [M + H-H_2_O]^+^ at *m*/*z* 457, [caffeoyltartaric acid-H_2_O]^+^ at *m*/*z* 295, and ions from the caffeoyl moiety ([caffeic acid + H]^+^ at *m*/*z* 181, [caffeic acid + H − H_2_O]^+^ at *m*/*z* 163, [caffeic acid + H − 2H_2_O]^+^ at *m*/*z* 145, and [caffeic acid + H − CO_2_]^+^ at *m*/*z* 135) supported that peak 3 is 2,3-DCTA. The TIC chromatogram extracted at *m*/*z* 517 yielded potassium adduct at *m*/*z* 555, sodium adduct at *m*/*z* 539, and a base peak at *m*/*z* 499 ([M + H − H_2_O]^+^) with 100% relative abundance indicating the identity of peak 7 to be 3,5-di-*O*-caffeoylquinic acid. Other fragment ions from the caffeoyl moiety ([caffeic acid + H − H_2_O]^+^ at *m*/*z* 163, [caffeic acid + H − 2H_2_O]^+^ at *m*/*z* 145 and [caffeic acid + H − CO_2_]^+^ at *m*/*z* 135), which are the MS fragmentation properties of 3,5-DCQA, were also observed. The MS spectra of the compounds identified are presented in a Supplementary File (Figure S5). All the hydroxycinnamoyl derivatives identified in this study were previously reported from red pigmented lettuce [2,8,25]. 

The concentrations of 3-CQA, 5-CQA, 3,5-DCQA, and 2,3-DCTA in lettuce germplasm collection and commercial cultivars were presented in Table S1. 2,3-DCTA (337.1 to 19,957.2 μg/g DW), commonly known as chicoric acid, was dominant in lettuce samples which contributed 35.4 to 91.5% of the total hydroxycinnamoyl derivatives determined, followed by 5-CQA (20.8 to 15,500.4 μg/g DW) and 3,5-DCQA (16.2 to 2217.8 μg/g DW). Caffeoylquinic acids (CQAs) and caffeoyltartaric acids (CTAs) are plant derived metabolites that help defense against stress to the plant [53] and posses wide ranges of therapeutic applications in humans [21,22,23]. The CQAs are ubiquitous in various plant-derived foods. Coffee is among the major sources of CQAs (total mono CQAs reported in the range between 45.79 to 1662.01 mg/L [54] and 40,360 and 61,720 μg/g [52]). The total hydroxycinnamoyl derivatives obtained in this study (403.3–31,351.3 μg/g DW) corroborates previous report of kim et al. (2019) 2654.0–45,803.0 μg/g DW) [7]. Other authors have also reported high amount of caffeic acid/hydrocinnamic acid derivatives in various lettuce samples. For example, Llorach et al. (2008) [32] determined total caffeic acid derivatives in the range between 17.1 (iceberg) and 203.0 (lollo rosso) mg/100g fresh weight while Nicolle et al. (2004) [55] reported hydroxycinnamic acids (8.4 to 27.8 mg/g), dicaffeoyltartaric acid (4.28 to 11.2 mg/g), and chlorogenic acid (0.2 to 6.1 mg/g DW). Cheng et al. (2014) [56] reported relatively high chlorogenic acid concentrations (23.9 to 26.8 mg/g DW) from red pigmented scarlet lettuce lines. Thus, the results of this study and previous reports suggest that lettuce could be regarded as among the major sources of the hydroxycinnamoyl derivatives, which are known for their high antioxidant properties [22]. As lettuce is increasingly being utilized in the human diet, quantitative data of CQAs and CTAs in lettuce helps to improve the estimates of daily intake of hydroxycinnamic acids and understand their bioavailability and bioactivities.

To study the effects of phenotypic characters on the concentrations of chemical components, average values of concentration of the compounds in samples with similar phenotypic characters and Duncan’s significant test between groups were computed. Significant differences were detected among various groups (Table 1). For example, the mean value of total hydroxycinnamoyl derivatives of dark + very dark red pigmented samples is 170% higher than that of light + very light red-pigmented accessions. Samples with broad obtrullate leaf shape had significantly lower mean concentration of hydroxycinnamoyl derivatives compared to samples with a medium elliptic, broad elliptic, and circular leaf shapes. Circular-shaped leaf samples had significantly higher average concentrations of 5-CQA and 2,3-DCTA compared to other shapes. Likewise, in anthocyanins, samples with a strong degree of undulation of the leaf margin showed significantly higher mean concentrations of 5-CQA, 2,3-DCTA, and 3,5-DCQA compared to weak and medium undulated leaf samples. The data for the effect of density of incisions on the leaf margin of the apical part are inconsistent. 

#### 3.3.3. Flavone and Flavonols

The flavonoids detected and identified using our instrument were conjugates of quercetin, luteolin, and cyanidin. The flavonols, quercetin 3-*O*-glucuronide, and quercetin 3-*O*-(6″-malonyl)glucoside, were the major flavonols of red pigmented lettuce in this study. The flavone luteolin 7-*O*-glucuronide was also detected in appreciable amounts. In addition to comparison with the UV maxima and retention times to those of authentic standards, the identification of flavonoids was further supported by the MS fragmentation data (Figure S5). The [M + H]^+^ at *m*/*z* 479, [M + Na]^+^ at *m*/*z* 501, [M + K]^+^ at *m*/*z* 517, and protonated quercetin aglycone ([quercetin + H]^+^) at *m*/*z* 303 that indicate the loss of a glucuronic residue (*m*/*z* 176) indicate the identity of peak 4 (Figure S4) to be quercetin 3-*O*-glucuronide. The [quercetin + H]^+^ was also presented in peak 6 (Figure S4) in addition to the base peak at *m/z* 551, and other minor peaks at *m*/*z* 573 and *m*/*z* 589 which are the characteristic properties of the protonated molecular ion of quercetin 3-*O*-(6″-malonyl)-glucoside, an adduct of Na, and an adduct of K, respectively. Peak 5 (Figure S4) presented a major peak at *m*/*z* 463 (100% relative abundance) that represents a protonated luteolin 7-*O*-glucuronide, and fragment ion at *m*/*z* 287 due to loss of glucuronide (*m*/*z* 176). 

As in the case of other metabolites described in previous sections, the effects of phenotypic characters on the levels of quercetin 3-*O*-glucuronide, quercetin 3-*O*-(6″-*O*-malonyl)glucoside, and luteolin 7-*O*-glucuronide were studied (Table 1). The mean quercetin 3-*O*-(6″-*O*-malonyl)glucoside and luteolin 7-*O*-glucuronide values of dark + very dark and medium red-pigmented leaf samples were significantly higher than the light + very light samples, indicating an increase in the concentration of these flavonoids with the intensity of the red color of the leaves. The circular-shaped leaves were found to accumulate the highest total flavonoids (flavones and flavonols) concentrations, followed by broad elliptic, medium elliptic, and broad obtrullate shaped leaves, in decreasing order. The mean values of total flavonoids (flavones and flavonols) of lettuce samples with strong degrees of undulation of leaf margin (11,041.0 μg/g DW) were significantly higher than those with medium (6382.8 μg/g DW) and weak (4582.4 μg/g DW) degrees of undulation. The contents of both flavones and flavonols were also found to increase with the density of incision of the leaf margin in the order of sparse, dense, and very dense. 

The concentrations of quercetin 3-*O*-glucuronide, quercetin 3-*O*-(6″-malonyl)glucoside, and luteolin 7-*O*-glucuronide of lettuce samples are presented in Table S2. The concentrations of flavonoids were significantly varied among lettuce samples. The sum of flavonoids (quercetin 3-*O*-glucuronide, quercetin 3-*O*-(6″-*O*-malonyl)glucoside, and luteolin 7-*O*-glucuronide) content ranged from 130.6 to 41,242.4 μg/g DW. Quercetin 3-*O*-(6″-malonyl)-glucoside was the most dominant flavonoid and ranged between 45.4 and 31,121.0 μg/g DW. Kim et al. (2019) [7] recently reported a concentration of quercetin 3-*O*-(6″-malonyl)glucoside from 220.0 (head lettuce) to 20,209.0 (red romaine lettuce) μg/g DW, and Dupont et al. (2000) [57] reported a 0.2 to 95.7 μg quercetin equivalent per gram of fresh weight (QE μg/g FW). Our results are slightly higher than the reports of Kim et al. (2019) [7] and Dupont et al. (2000) [57]. However, it should be noted that those authors analyzed small lettuce populations.

### 3.4. Multivariate Analysis

To study the statistical relationship between the biochemical characters, Pearson’s correlation analysis was performed using SPSS. The correlation analysis revealed highly significant correlations (*p* < 0.01) between the biochemicals and antioxidant activity. The strongest correlations were C3-3″MG vs. C3-6″MG, 2,3-DCTA vs. TPC, TPC vs. ABTS, 2,3-DCTA vs. ABTS, 3-CQA vs. 2,3-DCTA, and 5-CQA vs. 3,5 DCQA. The high correlations of biochemical characters with antioxidant activity support previous reports elsewhere [22,52]. The detailed correlation results are presented in Table 2. 

**Figure 3:**
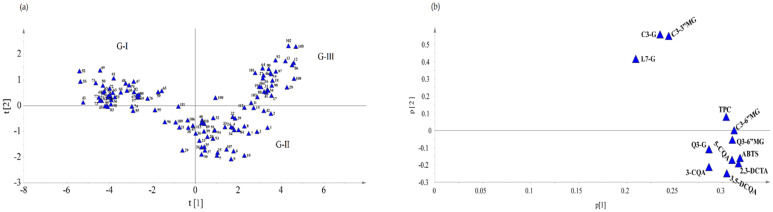
PCA score (**a**) and loading (**b**) plots of lettuce samples and metabolites. The first and second principal components contributed 77.2 and 8.4% of the total variations, respectively.

**Figure 4:**
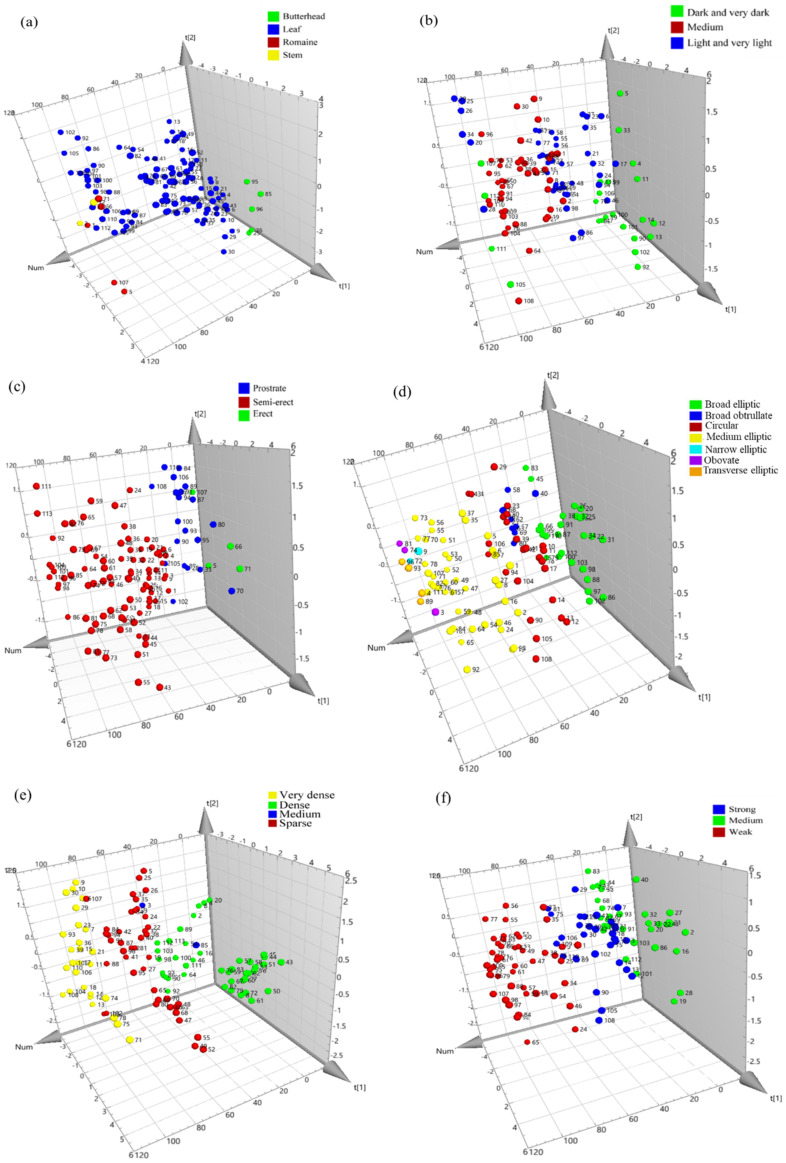
3D PLS-DA score plots of lettuce samples. The score plots were grouped based on their phenotypic characters: plant growth type (**a**); intensity of red color of the outer leaves (**b**); leaf attitude (**c**); leaf shape (**d**); leaf blade—density of incisions on margin on apical part (**e**); and leaf blade—degree of undulation of leaf margin (**f**). t[1] shows the variation between the groups, whereas t[2] captures the variation within the group.

A non-supervised multivariate analysis (principal components analysis, PCA) was used to investigate similarities and/or differences between lettuce samples. PCA scatter and loading plots of lettuce samples and metabolites are presented in Figure 3. The first two principal components explained 85.6% of the variations: the first (t[1]) and the second (t[2]) components contributed 77.2% (eigenvalue 9.27) and 8.4% (eigenvalue 1.01), respectively. In the PCA, groupings of lettuce samples into three major groups were observed located mainly at the left top quadrant (G-I, −t[1] and +t[2]), bottom right quadrant (G-II, +t[1] and −t[2]), and right top quadrant (G-III, +t[1] and +t[2]) of the scatter plot (Figure 4a). The loading plot (Figure 4b) showed that all the metabolites contributed positively to t[1], whereas only L7-G, C3-G, and C3-3″MG had a positive contribution to t[2]. Assessment of the loading and scatter plots indicated that the first group of lettuce samples (G-I) was characterized by the lowest concentrations of all metabolites; G-II by the lowest levels of L7-G, C3-3″MG, and C3-G, and intermediate levels of other metabolites; the last group (G-III) contained lettuce samples with the highest concentrations of most of the metabolites.

**Figure 5:**
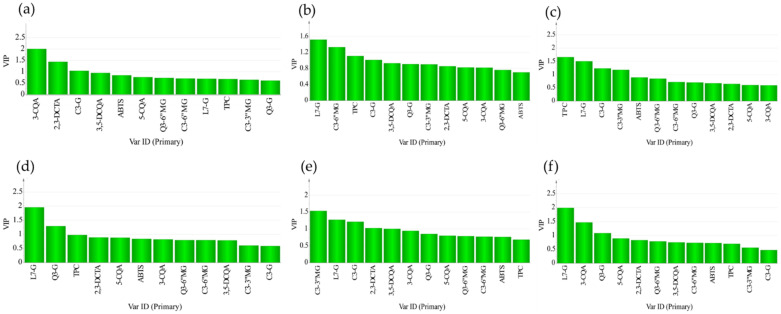
Variable importance in projection (VIP) values associated with the PLS-DA score plots grouped based on plant growth type (**a**); intensity of red color of the outer leaves (**b**); leaf attitude (**c**); leaf shape (**d**); leaf blade: density of incisions on the margin on apical part (**e**); and leaf blade: degree of undulation of the leaf margin (**f**).

Supervised partial least square discriminant analysis (PLS-DA) was used to assess whether correlations existed among the various phenotypic properties of the samples and their biochemical composition profiles. PLS-DA classified our lettuce samples into known groups, and the key variables that drive the classification were determined. Figure 4 shows the 3D score plots of lettuce samples which were classified based on their phenotypic properties. We attempted to discriminate 113 samples based on various phenotypic characters, such as plant growth type, the intensity of the red color of the outer leaves, leaf attitude, leaf shape, leaf blade (density of incisions on the margin on the apical part), and leaf blade (degree of undulation of the leaf margin). As shown in Figure 4, the 3D PLS-DA scatter plots allowed clear visualization of lettuce samples. Lettuce samples tended to show differences between groups and showed visible associations within each group (phenotypic characters). However, the variations within groups were also significantly high in some cases. The main variables (metabolites) that drive the variations presented in variable importance in projection (VIP) values (Figure 5) were 3-CQA, TPC, and C3-3″MG for the groups based on plant growth type, leaf attitude, and leaf blade (density of incisions on the margin on the apical part), respectively. L7-G was the major variable that expressed the difference in the classifications based on the intensity of the red color of the outer leaves, leaf shape, and leaf blade (degree of undulation of the leaf margin).

## 4. Conclusions

This study on profiles of biochemicals in red pigmented lettuce samples identified and quantified three anthocyanins (cyanidin 3-*O*-glucoside, cyanidin 3-*O*-(3″-*O*-malonyl)glucoside, and cyanidin 3-*O*-(6″-*O*-malonyl)glucoside), four hydroxycinnamoyl derivatives (3-*O*-caffeoylquinic acid, 5-*O*-caffeoylquinic acid, 3,5-di-*O*-caffeoylquinic acid, and 2,3-di-*O*-caffeoyltartaric acid), two flavonols (quercetin 3-*O*-glucuronide and quercetin 3-*O*-(6″-*O*-malonyl)glucoside, and a flavone (luteolin 7-*O*-glucuronide). In addition, the TPC and antioxidant activity were evaluated. Huge variation in biochemical characters among the lettuce samples was observed. Cyanidin 3-*O*-(6″-*O*-malonyl)glucoside, 2,3-di-*O*-caffeoyltartaric acid, and quercetin 3-*O*-(6″-*O*-malonyl)glucoside were the most dominant in red pigmented lettuce samples among the groups of metabolites considered in this study. Several reasons could contribute to the large variations that occurred in phytochemical contents in lettuce, including genotype and variety. In this study, the intensity of red color, leaf shape, plant growth type, density of incision, and degree of undulation of the leaf margin were also found to affect the concentrations of metabolites. Lettuce plants with high intensity of red in leaves, circular leaf shapes, a strong degree of leaf undulation, and highly dense leaf incisions were found to accumulate the highest concentration of flavonoids and hydroxycinnamic acids. In general, this study showed that red lettuce could be among the major dietary sources of antioxidants such as caffeic acid and quercetin derivatives. In addition, this study confirmed that consumers, breeders, and nutraceutical companies should also consider the phenotypic characters described above while choosing nutrient dense lettuce plants for various applications.

## Figures and Tables

**Table 1:** Effects of selected phenotypic characters of lettuce on the metabolite profile (*n* = number of lettuce samples).

The Intensity of Red Color of the Outer Leaves									
	Hydroxycinnamoyl derivatives (μg/g DW)					Flavone and flavonols (μg/g DW)			
	3-CQA	5-CQA	2,3-DCTA	3,5-DCQA	Total	Q3-G	L7-G	Q3-6″MG	Total
Very light + light (*n* = 48)	163.6 ^a^	2251.7 ^a^	4464.1 ^a^	355 ^a^	7234.4 ^a^	1979.9 ^a^	246.3 ^a^	2289.4 ^a^	4515.6 ^a^
Medium (*n* = 42)	199.9 ^a^	5069.5 ^b^	6983.3 ^b^	695.1 ^b^	12,947.7 ^b^	3092.7 ^a^	305.5 ^a^	4636.7 ^b^	8034.9 ^b^
Dark + very dark (*n* = 23)	217.4 ^a^	4675.6 ^b^	6740.9 ^ab^	635.5 ^b^	12,269.5 ^b^	3265.1 ^a^	488.9 ^b^	6029.7 ^b^	9783.6 ^b^
	Anthocyanins (μg/g DW)				ABTS	TPC	Leaf length	Leaf width	Plant weight
	C3-G	C3-3″MG	C3-6″MG	Total					
Very light + light (*n* = 48)	24.2 ^a^	5.5 ^a^	291.4 ^a^	321.1 ^a^	41,942.6 ^a^	44,529.4 ^a^	25.0 ^a^	19.0 ^a^	299.9 ^a^
Medium (*n* = 42)	38.5 ^ab^	11.7 ^a^	630.5 ^a^	680.8 ^a^	58,459.9 ^b^	57,758.4 ^b^	26.7 ^a^	20.5 ^a^	319.1 ^a^
Dark + very dark (*n* = 23)	65.0 ^b^	36.1 ^b^	1224 ^b^	1325.1 ^b^	61,652.6 ^b^	57,428.5 ^b^	25.5 ^a^	19.2 ^a^	322.3 ^a^
**Leaf Shape**									
	Hydroxycinnamoyl derivatives (μg/g DW)					Flavone and flavonols (μg/g DW)			
	3-CQA	5-CQA	2,3-DCTA	3,5-DCQA	Total	Q3-G	L7-G	Q3-6″MG	Total
Medium elliptic (*n* = 43)	182.6 ^b^	3139.9 ^b^	5145.2 ^b^	503.8 ^b^	8971.5 ^b^	1927.5 ^ab^	369.4 ^ab^	3278.7 ^ab^	5575.6 ^ab^
Broad elliptic (*n* = 27)	207.9 ^b^	4120.0 ^ab^	6562.2 ^bc^	597.6 ^b^	11,487.7 ^bc^	3228.7 ^bc^	272.6 ^ab^	3864.4 ^b^	7365.7 ^bc^
Circular (*n* = 25)	228.1 ^b^	5840.3 ^c^	8432.1 ^c^	723.4 ^b^	15,223.9 ^c^	4429.1 ^c^	407.9 ^b^	6846.5 ^c^	11,683.5 ^c^
Broad obtrullate (*n* = 9)	53.9 ^a^	594.9 ^a^	947.9 ^a^	92.4 ^a^	1689.1 ^a^	628.0 ^a^	117.2 ^a^	739.0 ^a^	1484.2 ^a^
	Anthocyanins (μg/g DW)				ABTS	TPC	Leaf length	Leaf width	Plant weight
	C3-G	C3-3″MG	C3-6″MG	Total					
Medium elliptic (*n* = 43)	34.6 ^ab^	8.3 ^ab^	453.4 ^ab^	496.3 ^ab^	47,204.0 ^b^	50,143.3 ^b^	27.3 ^b^	17.3 ^a^	295.6 ^a^
Broad elliptic (*n* = 27)	45.0 ^ab^	18.9 ^ab^	739.2 ^b^	803.0 ^b^	58,892.8 ^bc^	54,236.4 ^b^	27.7 ^b^	19.1 ^a^	330.8 ^a^
Circular (*n* = 25)	61.7 ^b^	27.8 ^b^	998.1 ^b^	1087.6 ^b^	64,500.1 ^c^	62,395.0 ^b^	21.7 ^a^	22.7 ^b^	297.7 ^a^
Broad obtrullate (*n* = 9)	3.6 ^a^	0.0 ^a^	36.9 ^a^	40.5 ^a^	22,470.2 ^a^	29,089.1 ^a^	23.7 ^ab^	22.8 ^b^	307.3 ^a^
**Leaf-Blade: Degree of Undulation of Margin**									
	Hydroxycinnamoyl derivatives (μg/g DW)					Flavone and flavonols (μg/g DW)			
	3-CQA	5-CQA	2,3-DCTA	3,5-DCQA	Total	Q3-G	L7-G	Q3-6″MG	Total
Weak (*n* = 48)	175.5 ^a^	2078.6 ^a^	4374.6 ^a^	344.7 ^a^	6973.4 ^a^	1750.6 ^a^	316.7 ^a^	2515.2 ^a^	4582.4 ^a^
Medium (*n* = 34)	169.3 ^a^	3999.4 ^b^	5594.5 ^a^	586.5 ^b^	10,349.6 ^a^	2575.6 ^a^	259.7 ^a^	3547.5 ^a^	6382.8 ^a^
Strong (*n*= 31)	228.0 ^a^	6218.9 ^c^	8465.3 ^b^	785.9 ^b^	15,698.2 ^b^	4143.0 ^b^	382.8 ^a^	6515.2 ^b^	11,041.0 ^b^
	Anthocyanins (μg/g DW)				ABTS	TPC	Leaf length	Leaf width	Plant weight
	C3-G	C3-3″MG	C3-6″MG	Total					
Weak (*n* = 48)	21.9 ^a^	6.0 ^a^	325.4 ^a^	353.3 ^a^	42,626.3 ^a^	43,499.4 ^a^	27.5 ^b^	17.4 ^a^	344.6 ^b^
Medium (*n* = 34)	39.1 ^ab^	11.9 ^a^	598.5 ^a^	649.5 ^a^	53,055.6 ^a^	52,732.3 ^a^	27.1 ^b^	19.9 ^b^	301.1 ^ab^
Strong (*n*= 31)	61.2 ^b^	28.9 ^b^	1053.3 ^b^	1143.3 ^b^	65,697.3 ^b^	64,621.0 ^b^	21.6 ^a^	22.7 ^c^	275.6 ^a^
**Leaf-Blade: Density of Incisions on the Margin on the Apical Part**									
	Hydroxycinnamoyl derivatives (μg/g DW)					Flavone and flavonols (μg/g DW)			
	3-CQA	5-CQA	2,3-DCTA	3,5-DCQA	Total	Q3-G	L7-G	Q3-6″MG	Total
Sparse (*n* = 42)	222.8 ^b^	3344.0 ^a^	6389.5 ^ab^	601.9 ^b^	10,558.2 ^ab^	2199.0 ^a^	305.1 ^a^	3459.5 ^a^	5963.6 ^a^
Dense (*n* = 42)	144.1 ^a^	3046.6 ^a^	4285.4 ^ab^	362.8 ^a^	7838.9 ^a^	2488.2 ^ab^	332.4 ^a^	3439.1 ^a^	6259.7 ^ab^
Very dense (*n* = 27)	200.6 ^ab^	5520.4 ^b^	7388.2 ^b^	696.2 ^b^	13,805.4 ^b^	3727.2 ^b^	327.4 ^a^	5508.1 ^a^	9562.7 ^b^
	Anthocyanins (μg/g DW)				ABTS	TPC	Leaf length	Leaf width	Plant weight
	C3-G	C3-3″MG	C3-6″MG	Total					
Sparse (*n* = 42)	46.1 ^a^	13.7 ^a^	546.1 ^a^	605.9 ^a^	54,766.2 ^ab^	52,468.3 ^ab^	27.6 ^b^	18.1 ^a^	306.6 ^a^
Dense (*n* = 42)	35.1 ^a^	12.4 ^a^	586.2 ^a^	633.6 ^a^	44,272.3 ^a^	47,073.9 ^ab^	26.5 ^b^	19.3 ^a^	335.3 ^a^
Very dense (*n* = 27)	32.2 ^a^	17.7 ^a^	735.9 ^a^	785.8 ^a^	60,146.6 ^b^	58,655.7 ^b^	21.9 ^a^	22.2 ^b^	282.3 ^a^

Different letters within the same column denote significant differences (*p* < 0.05). The ABTS radical scavenging activity and TPC are expressed as μgTE/g DW and μgGAE/g DW, respectively. The leaf length and leaf width are expressed in centimeters (cm) and plant weight in grams (g). 3-CQA, 3-*O*-caffeoylquinic acid; 5-CQA, 5-*O*-caffeoylquinic acid; 2,3-DCTA, 2,3-di-*O*-caffeoyltartaric acid; 3,5-DCQA, 3,5-di-*O*-caffeoylquinic acid; Q3-G, quercetin 3-*O*-glucuronide; L7-G, luteolin 7*-O*-glucuronide; Q3-6″MG, quercetin 3-*O*-(6″-*O*-malonyl)glucoside; C3-G, cyanidin 3-*O*-glucoside; C3-3″MG; cyanidin 3-*O*-(3″-*O*-malonyl)glucoside; C3-6″MG, cyanidin 3-*O*-(6″-*O*-malonyl)glucoside; ABTS, 2,2′-azinobis-(3-ethylbenzothiazoline-6-sulfonic acid) radical scavenging activity; TPC, total phenolic content.

**Table 2:** Pearson’s correlations among the hydroxycinnamoyl derivatives, flavonoids, total phenolic content, and ABTS radical reducing potential.

	3-CQA	5-CQA	2,3-DCTA	3,5-DCQA	Q3-G	L7-G	Q3-6″MG	C3-G	C3-3″MG	C3-6″MG	ABTS
5-CQA	0.488										
2,3-DCTA	0.881	0.786									
3,5-DCQA	0.604	0.839	0.812								
Q3-G	0.544	0.551	0.649	0.431							
L7-G	0.334	0.416	0.392	0.369	0.434						
Q3-6″MG	0.499	0.699	0.686	0.596	0.707	0.798					
C3-G	0.380	0.518	0.526	0.384	0.503	0.366	0.549				
C3-3″MG	0.340	0.591	0.538	0.415	0.431	0.437	0.646	0.753			
C3-6″MG	0.447	0.748	0.668	0.571	0.539	0.526	0.770	0.700	0.946		
ABTS	0.760	0.777	0.876	0.770	0.665	0.400	0.663	0.486	0.441	0.601	
TPC	0.741	0.893	0.926	0.822	0.645	0.425	0.688	0.574	0.599	0.751	0.892

All correlations are significant at the 0.01 level (2-tailed); color scale: 1 (green) and −1 (red) indicate the highest positive and negative correlations, respectively. 3-CQA, 3-*O*-caffeoylquinic acid; 5-CQA, 5-*O*-caffeoylquinic acid; 2,3-DCTA, 2,3-di-*O*-caffeoyltartaric acid; 3,5-DCQA, 3,5-di-*O*-caffeoylquinic acid; Q3-G, quercetin 3-*O*-glucuronide; L7-G, luteolin 7-*O*-glucuronide; Q3-6″MG, quercetin 3-*O*-(6″-*O*-malonyl)glucoside; C3-G, cyanidin 3-*O*-glucoside; C3-3″MG; cyanidin 3-*O*-(3″-*O*-malonyl)glucoside; C3-6″MG, cyanidin 3-*O*-(6″-*O*-malonyl)glucoside; ABTS, 2,2′-azinobis-(3-ethylbenzothiazoline-6-sulfonic acid) radical scavenging activity; TPC, total phenolic content.

## Data Availability

Data will be available on request to the authors.

## Supplementary Materials

The following are available online at https://www.mdpi.com/article/10.3390/foods10102504/s1. Figure S1: External standard calibration curves of hydroxycinnamoyl derivatives, flavone, and flavonols. Figure S2: Modified International Union for the Protection of new Varieties of Plants (UPOV) descriptions pictorial explanation for selected individual characteristics. Figure S3: A representative UPLC-PDA chromatogram of anthocyanins from lettuce extract (top) and cyanidin-3-*O*-glucoside standard (bottom) at 520 nm. Peaks identified as 1, cyanidin-3-*O*-glucoside; 2, cyanidin 3-*O*-(3″-*O*-malonyl)glucoside; 3, and cyanidin 3-*O*-(6″-*O*-malonyl)glucoside. Figure S4: A representative UPLC-PDA chromatogram of lettuce extract (top) and a mixture of standard compounds (bottom) at 350 nm. Peaks identified as 1, 3-*O*-cafffeoylquinic acid; 2, 5-*O*-caffeoylquinic acid; 3, 2,3 di-*O*-caffeoyltartaric acid; 4, quercetin 3-*O*-glucuronide; 5, luteolin 7-*O*-glucuronide; 6, quercetin 3-*O*-(6″-*O*-malonyl)glucoside; and 7, 3,5-di-*O*-caffeoylquinic acid. Figure S5: MS spectra in positive ion mode of hydroxycinnamoyl derivatives, flavones, and flavonols identified in lettuce samples. Table S1: Morphological characterization of lettuce (*Lactuca sativa* L.) made at the harvest maturity based on guidelines for the conduct of tests for distinctness, uniformity, and stability of modified International Union for the Protection of New Varieties of Plants (UPOV) descriptors. Table S2: The contents of metabolites from 113 germplasm collections and commercial cultivars of lettuce (*Lactuca sativa* L.) samples.
