# Supplementary material for: Nutritional Metabolites of Red Pigmented Lettuce (Lactuca sativa) Germplasm and Correlations with Selected Phenotypic Characters
Source: Foods. 2021 Oct 19;10(10):2504. doi: 10.3390/foods10102504 (PMC8535348; doi:10.3390/foods10102504)
Supplement: Supplementary file 1 [file foods-10-02504-s001.zip › foods-1412258-supplementary.pdf]

Nutritional Metabolites of Red Pigmented Lettuce (*Lactuca sativa*) Germplasm Collection and Correlations with Selected Phenotypic Characters

Awraris Derbie Assefa †, On-Sook Hur †, Bum-Soo Hahn, Bichsaem Kim, Na-Young Ro and Ju-Hee Rhee*

National Agrobiodiversity Center, National Institute of Agricultural Sciences, RDA, Jeonju 54874, Korea; awraris@korea.kr (A.D.A); oshur09@korea.kr (O.-S.H); bshahn@korea.kr (B.-S.H.); bsam92@korea.kr (B.K.); nonanona@korea.kr (N.-Y.R.)

* Correspondence: rheehk@korea.kr

† These authors contributed equally to this work.

Figure S1. External standard calibration curves of hydroxycinnamoyl derivatives, flavone, and flavonols

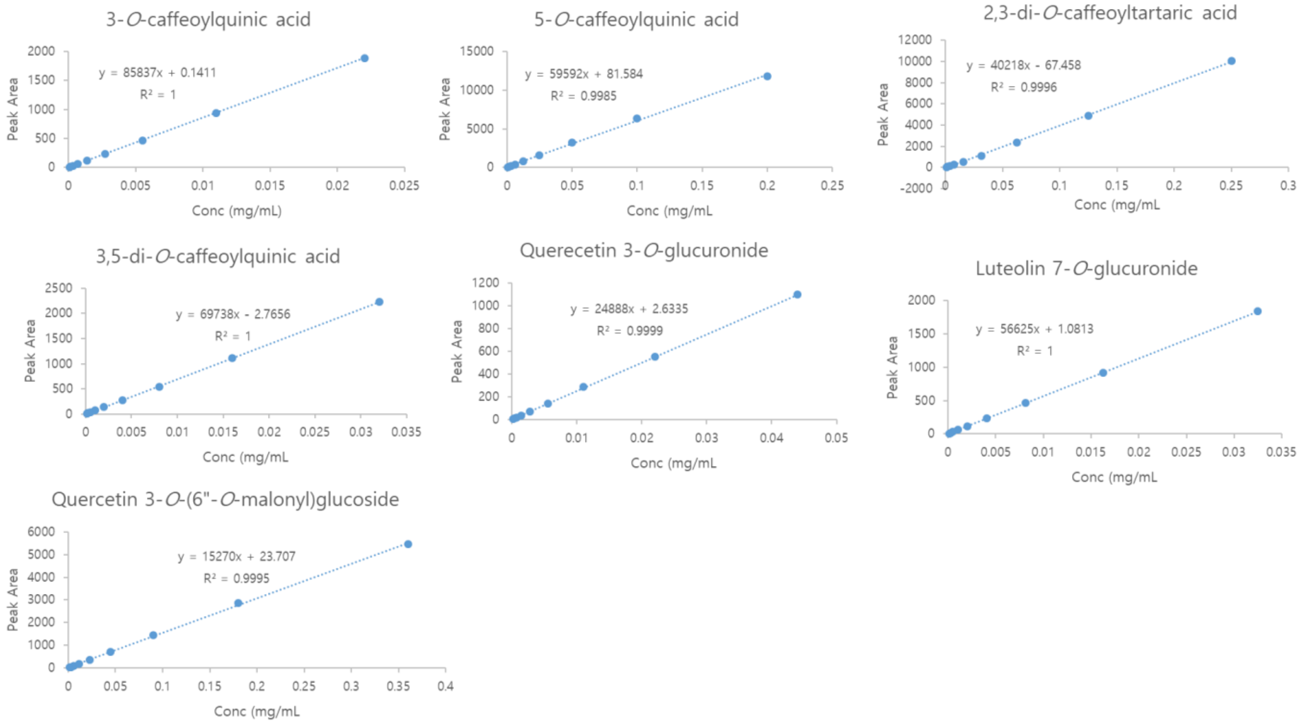

Figure S2. Modified International Union for the Protection of new Varieties of Plants (UPOV) descriptions pictorial explanation for selected individual characteristics (NB: Figures are adapted from UPOV, Guidelines for the conduct of tests for distinctness, uniformity and stability, www.upov.int accessed on July 30, 2021)

Plant Botanical name: *Lactuca sativa* L.

I. Plant growth type

A. Butterhead

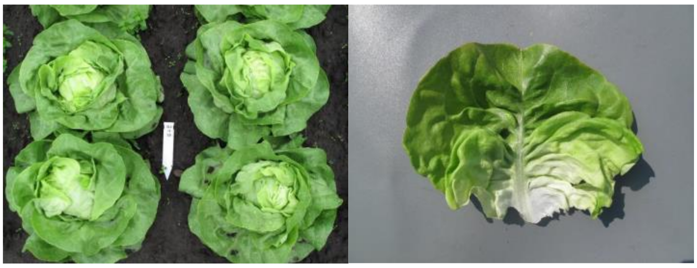

B. Cos/romaine

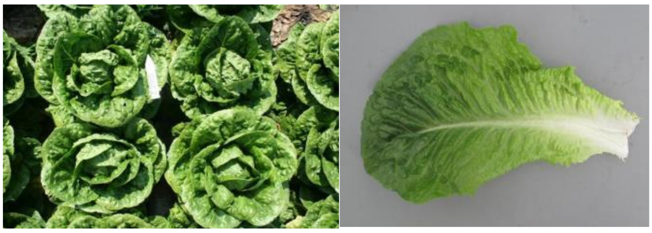

C. Leafy

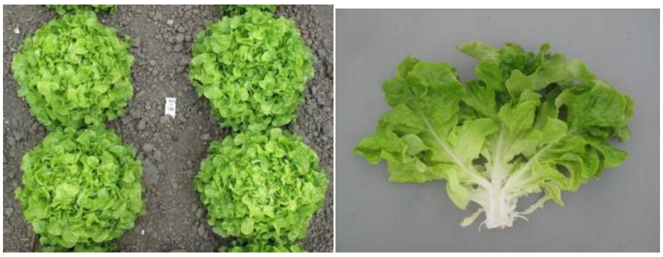

D. Stem

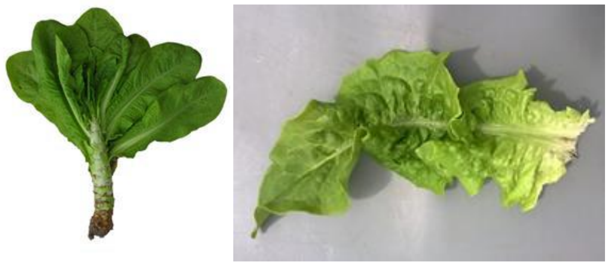

II. Leaf attitude

Erect	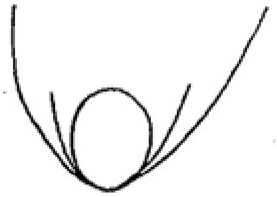
Semi erect	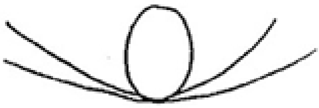
Prostrate/horizontal	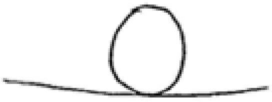

III. Leaf shape

Narrow elliptic	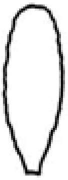	Circular	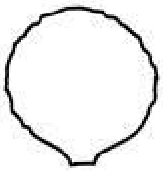
Medium elliptic	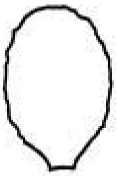	Obovate	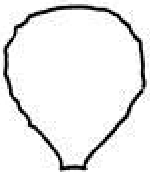

Broad elliptic	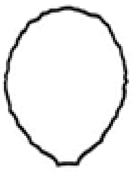	Broad obtrullate	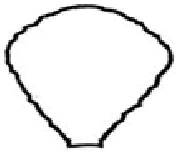
Transverse elliptic	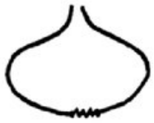		

Figure S3. A representative UPLC-PDA chromatogram of anthocyanins from lettuce extract (top) and cyanidin-3-*O*-glucoside standard (bottom) at 520 nm. Peaks identified as 1, cyanidin-3-*O*-glucoside; 2, cyanidin 3-*O*-(3″-*O*-malonyl)glucoside; 3, and cyanidin 3-*O*-(6″-*O*-malonyl)glucoside.

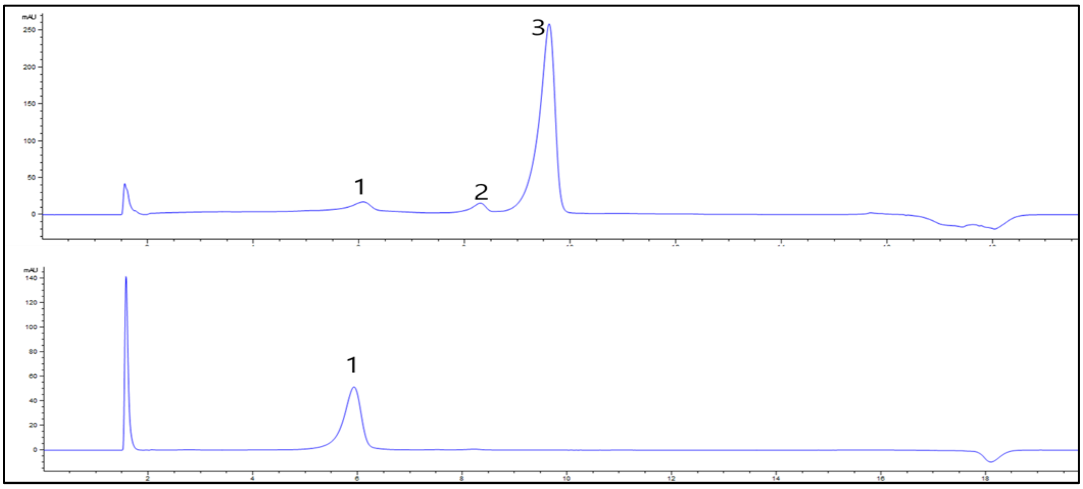

Figure S4. A representative UPLC-PDA chromatogram of lettuce extract (top) and a mixture of standard compounds (bottom) at 350 nm. Peaks identified as 1, 3-*O*-cafffeoylquinic acid; 2, 5-*O*-caffeoylquinic acid; 3, 2,3 di-*O*-caffeoyltartaric acid; 4, quercetin 3-*O*-glucuronide; 5, luteolin 7-O-glucuronide; 6, quercetin 3-*O*-(6''-*O*-malonyl)glucoside; and 7, 3,5-di-*O*-caffeoylquinic acid

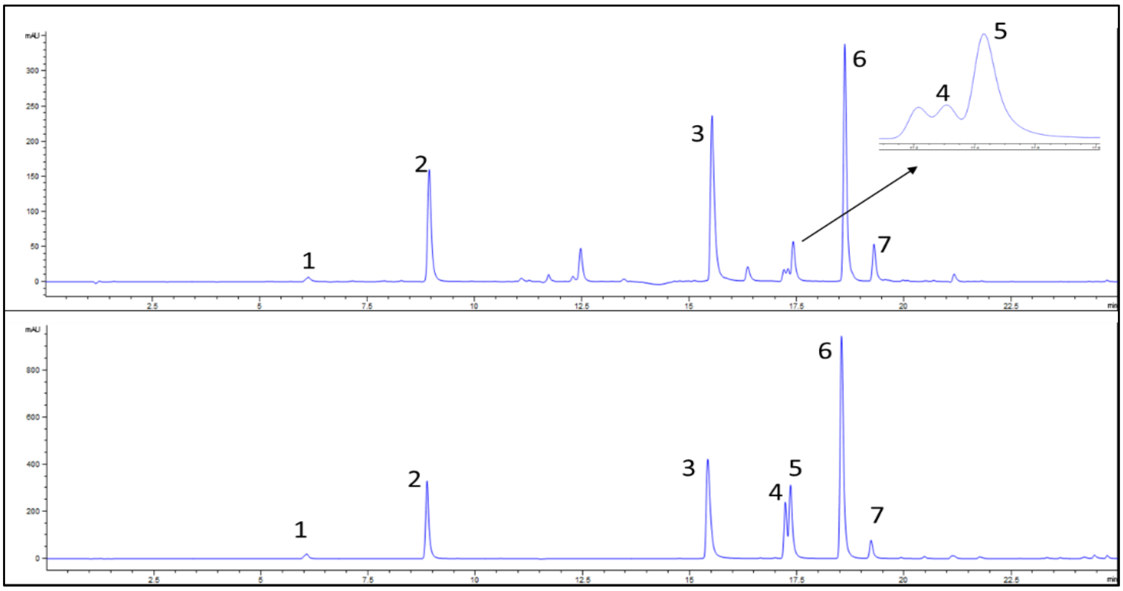

Figure S5. MS spectra in positive ion mode of hydroxycinnamoyl derivatives, flavones and flavonols identified in lettuce samples.

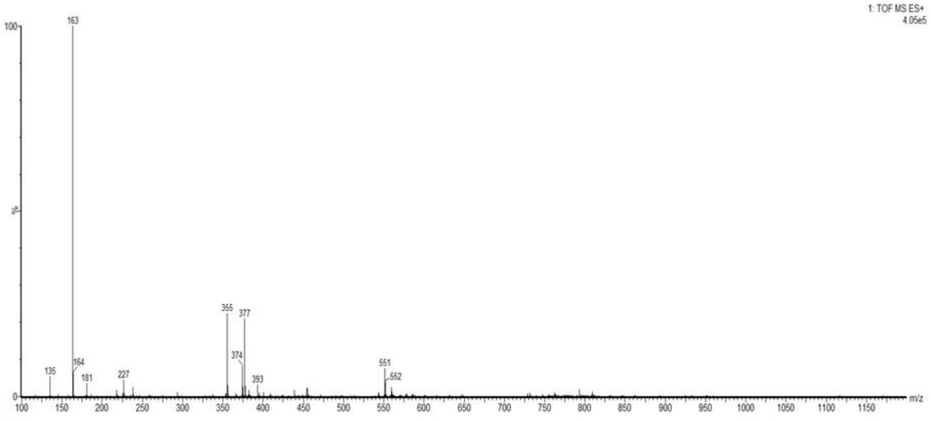

3-Caffeoylquinic acid

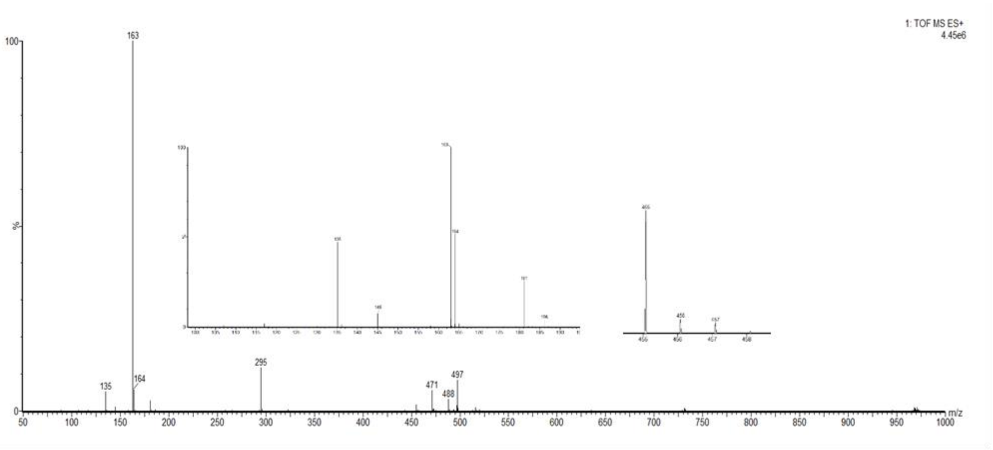

2,3 Dicaffeoyltartaric acid

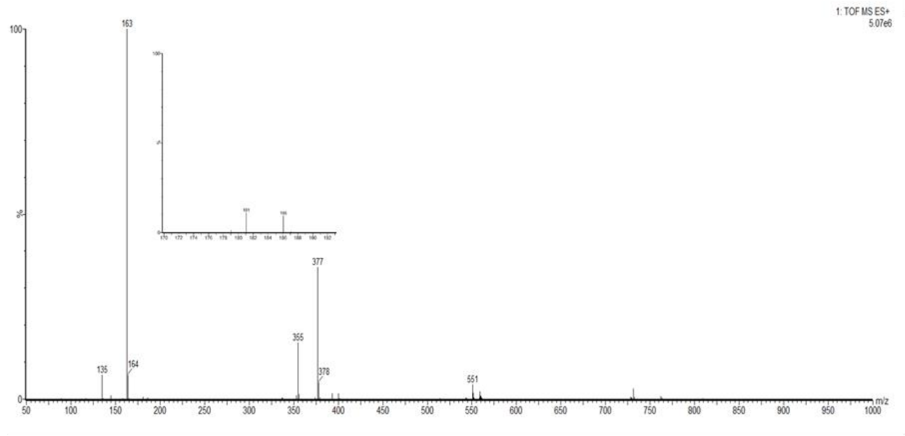

5-Caffeoylquinic acid

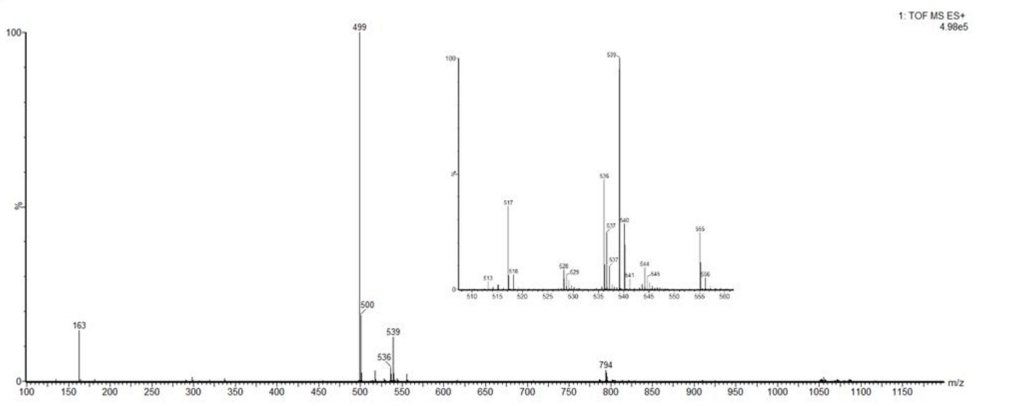

3,5 Dicaffeoylquinic acid

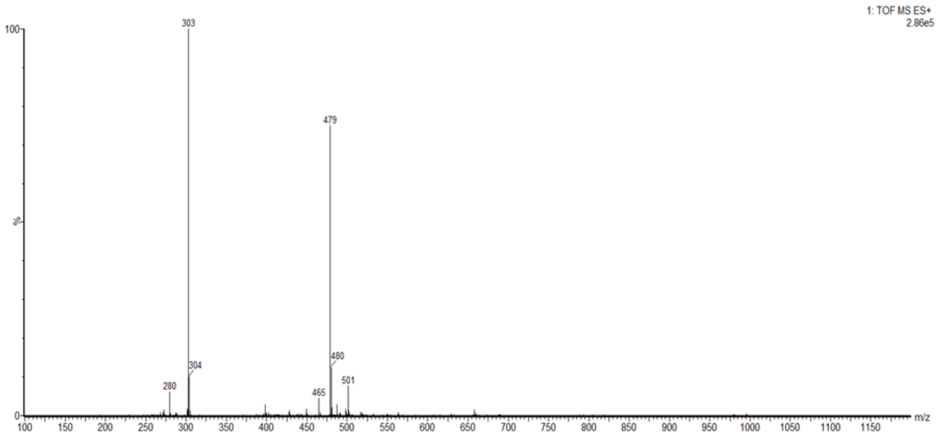

Quercetin 3-*O*-glucuronide

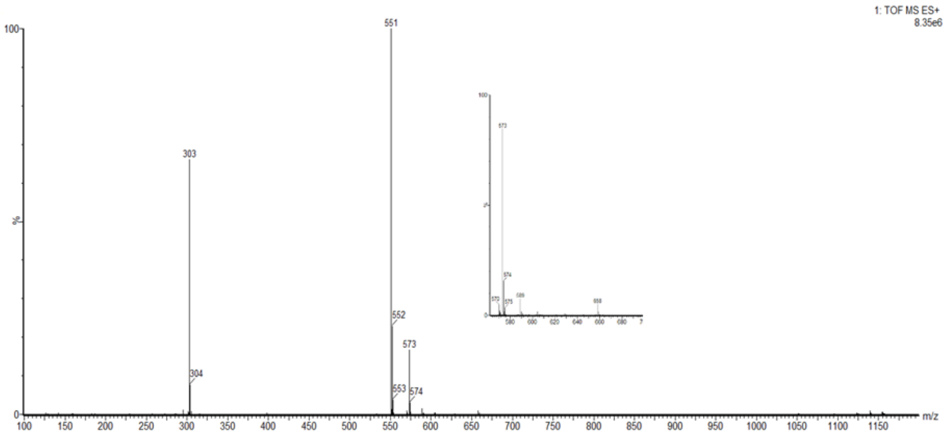

Quercetin 3-*O*-(6′′-malonyl)glucoside

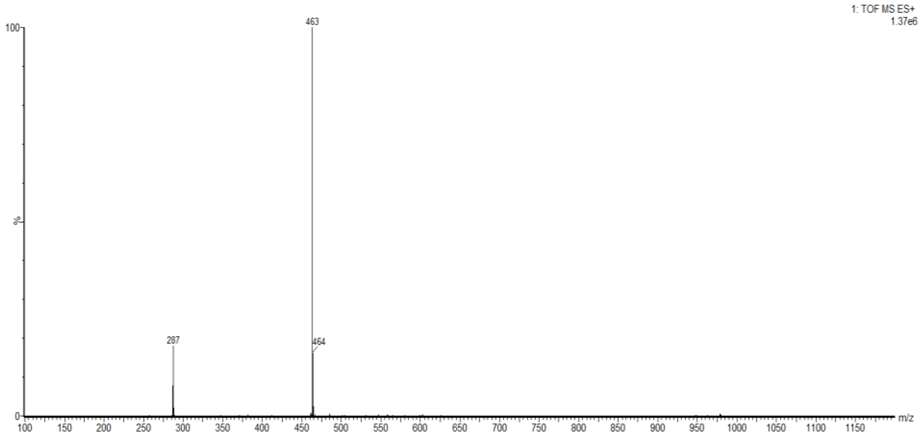

Luteolin 7-*O*-glucuronide

Table S1. Morphological characterization of lettuce (*Lactuca sativa* L.) made at the harvest maturity based on guidelines for the conduct of tests for distinctness, uniformity, and stability of modified International Union for the Protection of New Varieties of Plants (UPOV) descriptors.

S/No	IT No	CRC	PGT	IRCOL	LA	LS	LB: DUM	LB: DIMAP	LL* (cm)	LW* (cm)	PW* (g)
1	217012	1	3	3	2	2	1	1	30.5	16.3	235.0
2	218395	0	4	3	2	2	2	3	28.7	16.7	168.3
3	218396	0	2	3	2	6	3	2	23.3	21.5	166.7
4	219841	0	2	4	2	5	2	3	34.3	18.0	580.0
5	220010	0	3	4	1	2	1	1	33.7	15.8	290.0
6	228752	1	2	2	2	2	3	4	23.7	20.5	123.3
7	228753	1	2	3	2	2	3	4	21.2	23.0	165.0
8	228760	1	2	3	2	2	2	3	28.3	16.8	135.0
9	228871	1	2	3	2	1	3	4	23.3	20.7	190.0
10	231265	0	2	3	2	4	3	4	21.7	21.0	148.3
11	231521	0	2	4	2	4	3	4	18.5	20.5	173.3
12	231524	1	2	4	2	4	3	4	22.2	24.3	211.7
13	231525	1	2	4	2	4	3	4	22.0	20.7	153.3
14	231526	1	2	4	2	4	3	4	24.3	24.8	168.3
15	231527	0	2	3	2	4	3	4	23.8	20.7	255.0
16	231529	1	2	3	2	2	2	3	28.2	22.3	315.0
17	231531	0	2	2	2	4	3	4	21.3	23.0	293.3
18	231532	0	2	3	2	4	3	4	20.1	22.7	316.7
19	235353	0	2	3	2	2	2	1	16.0	8.7	68.3
20	242970	0	2	1	2	3	2	3	31.7	16.7	265.0
21	247142	1	2	2	2	4	3	4	24.7	26.0	230.0
22	251824	0	2	3	2	3	2	1	29.3	16.7	96.7
23	259307	1	2	2	2	4	3	4	13.5	15.8	65.0
24	260857	0	2	2	2	2	1	1	21.5	13.3	95.0
25	264962	0	1	1	2	3	1	1	18.3	12.3	226.7
26	264963	0	1	1	2	3	1	1	18.3	12.3	226.7
27	264964	0	2	3	2	2	2	1	27.2	15.6	186.7
28	264965	0	2	1	3	2	2	1	24.2	13.6	103.3
29	264970	0	2	1	2	4	3	4	21.2	22.7	250.0
30	264971	0	2	3	2	4	3	4	24.2	22.7	280.0
31	264972	0	2	3	2	3	2	1	97.1	21.8	201.7
32	264973	0	2	2	2	3	2	1	28.2	17.5	248.3
33	264974	0	2	4	3	3	2	1	28.8	23.6	265.0
34	267591	0	2	1	2	3	1	1	32.0	12.0	235.0
35	271099	0	2	2	2	2	1	1	27.7	18.2	445.0
36	271117	1	2	3	2	4	3	4	19.8	17.3	225.0
37	271118	0	2	2	2	2	1	1	42.5	17.2	430.0
38	271119	0	2	3	2	3	1	1	28.5	14.5	323.3
39	271120	0	2	3	2	4	3	4	22.7	23.5	280.0
40	271159	0	2	3	2	7	2	1	27.8	19.7	371.7
41	217005	0	2	3	2	4	3	1	22.8	23.8	301.7
42	276155	0	2	3	2	4	3	1	20.5	20.8	271.7
43	100511	0	2	1	2	4	2	3	27.8	26.2	641.7
44	100514	0	2	1	2	4	2	3	22.2	22.5	245.0
45	100516	0	2	1	2	3	2	3	20.5	20.7	261.7
46	101048	0	2	2	2	2	1	3	29.0	17.5	ND
47	101236	0	2	4	2	2	1	1	29.3	15.7	400.0
48	102664	0	2	2	2	2	1	1	32.0	16.3	270.0
49	102785	0	2	2	2	2	1	1	29.8	16.3	401.7
50	103152	0	2	3	2	2	1	3	30.2	15.0	301.7

51	104513	0	2	1	2	2	1	3	29.7	19.8	346.7
52	108868	0	2	2	2	2	1	1	24.8	23.8	295.0
53	110820	0	2	3	2	2	1	3	24.3	19.7	223.3
54	113396	0	2	2	2	2	1	3	24.0	17.5	ND
55	138082	0	2	2	2	2	1	1	33.0	17.8	395.0
56	178618	0	2	2	2	2	1	3	25.0	22.0	351.7
57	180492	0	2	2	2	2	1	3	24.7	21.0	258.3
58	181983	0	2	2	2	7	2	3	21.8	22.5	325.0
59	185751	0	2	4	2	2	1	3	29.8	12.8	200.0
60	195057	0	2	2	2	2	1	3	28.7	17.7	348.3
61	195058	0	2	2	2	2	1	3	25.5	25.0	485.0
62	203380	0	2	3	2	7	1	3	26.8	21.0	288.3
63	203381	0	2	3	2	7	1	1	24.0	26.0	388.3
64	204097	0	2	3	2	2	1	3	25.3	15.5	ND
65	206716	0	2	2	2	2	1	1	22.7	10.7	73.3
66	206800	1	3	2	1	3	1	1	30.0	16.8	355.0
67	206823	0	2	3	2	7	2	3	27.7	24.7	321.7
68	213458	0	2	2	2	7	2	1	20.0	19.3	198.3
69	213460	0	2	2	2	7	2	1	22.3	20.7	366.7
70	215799	0	2	1	3	2	1	1	25.2	13.2	318.3
71	215802	1	3	2	1	2	1	4	28.8	18.3	543.3
72	216990	0	4	3	2	1	1	3	45.5	11.2	448.3
73	217391	0	2	2	2	2	2	3	22.0	26.7	351.7
74	217504	0	2	2	2	6	2	4	19.3	16.5	105.0
75	217844	1	2	1	2	7	3	4	18.0	27.0	182.5
76	218010	1	2	3	2	2	1	3	32.7	17.0	495.0
77	218329	1	2	2	2	2	1	3	31.3	16.7	226.7
78	219838	0	2	3	2	2	1	4	26.0	16.5	358.3
79	219884	1	2	4	2	3	1	3	26.0	20.0	420.0
80	220036	1	2	3	3	7	2	1	25.0	24.7	323.3
81	220742	1	2	2	2	6	3	3	19.7	21.7	255.0
82	220743	1	2	4	2	2	2	3	24.3	15.0	230.0
83	251813	0	2	2	2	3	2	3	26.0	25.3	363.3
84	195215	0	2	2	3	2	1	1	33.3	15.5	395.0
85	213461	0	1	2	2	4	1	2	20.0	24.3	550.0
86	228749	1	2	2	2	3	2	3	23.0	19.0	236.3
87	251825	0	2	2	3	3	1	1	26.5	18.8	490.0
88	251826	0	2	3	3	3	1	1	27.8	18.5	510.0
89	262380	0	2	3	3	5	3	3	24.7	26.7	596.7
90	262402	0	2	4	3	4	3	3	22.3	24.3	333.3
91	262406	0	2	3	3	3	2	1	24.2	26.6	548.3
92	262407	0	2	4	2	2	1	3	30.7	18.7	541.7
93	262409	0	2	2	3	5	2	4	21.2	23.8	605.0
94	262411	0	2	3	3	4	3	1	24.5	28.2	596.7
95	271138	0	1	3	3	3	1	1	19.7	19.8	346.7
96	271139	0	1	3	2	5	1	1	18.8	22.9	378.3
97	271148	1	2	2	2	3	1	3	24.8	18.5	188.7
98	276099	0	2	2	2	3	1	3	20.5	12.3	128.0
99	280087	0	2	4	3	2	1	1	27.4	20.2	531.7
100	280088	0	2	4	3	3	1	3	27.2	17.5	533.3
101	296684	0	2	4	2	2	2	3	27.7	17.2	348.3
102	301289	1	2	4	3	3	3	1	25.7	24.7	331.7
103	301994	0	2	3	2	3	2	3	20.7	21.8	395.0
104	302032	0	2	3	2	4	2	4	24.2	25.5	443.3
105	302033	0	2	5	3	4	3	1	13.7	14.6	125.0
106	Seonpung plus **	0	2	4	3	4	3	4	23.0	26.5	438.0

107	Jeokromaine dessertrosa**	1	3	5	1	2	1	1	24.6	14.5	217.3
108	Jinbbal**	0	2	3	3	4	3	4	22.0	26.1	447.3
109	Nonghyeopheukchima**	0	2	3	3	3	3	4	19.1	23.8	412.0
110	Sinhong jeokchukmyeon**	0	2	3	3	3	3	4	21.6	24.6	558.0
111	Sambokmeokchima**	1	2	5	2	2	2	3	20.0	12.8	158.0
112	Power red romaine **	1	2	5	2	3	2	3	26.1	18.8	269.3
113	Superseonpung **	0	2	4	2	3	2	3	25.1	20.0	494.6

*Mean of 10 plants/leaves; ** Commercial cultivars; CRC = Cotyledon red color, 0 absent and 1 present; PGT = Plant growth type, 1 butterhead, 2 leaf, 3 romaine, and 4 stem; IRCOL = Intensity of red color of outer leaves, 1 very light, 2 light, 3 medium, 4 dark, and 5 very dark; LA = leaf attitude, 1 erect, 2 semi erect, and 3 prostrate; LS = Leaf shape, 1 narrow elliptic, 2 medium elliptic, 3 broad elliptic, 4 circular, 5 transverse elliptic, 6 obovate, and 7 broad obtrullate; LB:DUM = Leaf blade: degree of undulation of margin, 1 weak, 2 medium, and 3 strong; LB:DIMAP = Leaf blade: density of incisions on margin on apical part, 1 sparse, 2 medium, 3 dense, and 4 very dense; LL = Leaf length; LW = Leaf width; PW = Plant weight; ND = Not determined; IT NO = introduction number

Table S2. The contents of metabolites from 113 germplasm collection and commercial cultivars of lettuce (*Lactuca sativa* L.) samples

S/No	Hydroxycinnamoyl derivatives (μg/g DW)					Flavones and flavonols (μg/g DW)				Anthocyanins (μg/g DW)				ABTS (μg TE/g DW)	TPC (μgGAE/g DW)
	3-CQA	5-CQA	2,3-DCTA	3,5-DCQA	Total	Q3-G	L7-G	Q3-6"MG	Total	C3-G	C3-3"MG	C3-6"MG	Total		
1	532.1±18.7	5100.6±83.7	16718±2026.7	1055.4±19	23406.1	5701±158.2	131.8±2.3	8804.9±242	14637.7	ND	46.4±0.8	2385.9±45.2	2432.3	79428.7±190.6	89536.2±1265
2	229±18.9	15500.4±272.6	12303.1±1569.1	2111.8±44.2	30144.3	2693.5±362	350.5±15.2	7175.2±203.1	10219.2	ND	44.4±1.4	2303.3±40.6	2347.7	78365.9±143.6	102868.5±25.1
3	318.5±19.2	10718.7±310.3	12976.7±1617.4	1375.2±79.5	25389.1	2530.5±357.8	192.7±10	4394.1±130.5	7117.3	ND	13±0.6	1183.8±19.1	1196.8	79618.3±101.2	94161.4±1350.3
4	266.4±17.4	5832.5±133.5	6300.5±993.4	957.4±26.8	13356.8	1567±191.3	158.5±5.5	3172.4±85.1	4897.9	ND	10.8±0.2	1093±17.2	1103.8	72647.9±110.5	65220.4±329.6
5	438.9±20.4	1892.8±11.9	9587.2±949.9	898.6±46	12817.5	1520.9±82.3	74.1±0.6	1750.5±38.5	3345.5	ND	ND	341.2±9.1	341.2	71444.5±22.2	61066.7±236.8
6	249.7±13.7	7545.3±312	9405.4±1060.4	1285.3±82.5	18485.7	3321.5±27.6	148.8±4.7	3609.3±139.3	7079.6	ND	ND	371.2±15.2	371.2	74264.9±342.6	74054.2±27
7	152.2±10.9	10497.4±400.9	9260±1300.9	941.2±34.3	20850.8	7990.3±210.2	192.8±4.8	7758.1±304.6	15941.2	ND	5.8±0.6	956.8±9	962.6	76660.9±204.4	84596.2±219.2
8	185.4±19.7	10716±513.3	8105.7±1193.9	1018.7±82.4	20025.8	2150±258.7	243.6±14	5076.6±265.1	7470.2	ND	8.3±0.8	995.4±11.9	1003.7	77070.3±177.4	82546±305.2
9	403.5±47.5	5417.5±330.7	12019.1±1845	648.2±34	18488.3	2663.4±374.4	71.2±3.9	3923.5±272.6	6658.1	ND	ND	624.4±20.5	624.4	75184.8±110.4	72855.6±243.5
10	353.9±35	9984.9±569.7	13129.7±1812.8	1404.8±64	24873.3	2998.9±401.2	159.4±8.4	4199±246.7	7357.3	ND	ND	625.9±10	625.9	78748.6±234	81877.5±284.6
11	202.8±15.4	8055.1±409.4	7959.7±1019	892.1±49.8	17109.7	4610.9±550.2	226.2±17.9	6308.5±362.1	11145.6	28.3±0.6	20.7±0.7	977.0±16.1	1026	72669.9±314.7	68864.5±500.1
12	354.6±31.1	12301±412.3	15014.1±2158.1	1050±24.9	28719.7	4952.8±631.4	684.1±26.7	9393±381.2	15029.9	157.2±3.2	130.1±2.7	3432.2±49.3	3719.5	78510.6±262.7	97264±464.1
13	235.8±10.6	12950.6±216.4	12628.2±1392.6	994.6±32	26809.2	4319.4±198	763.1±24.3	9529.8±229.2	14612.3	145.1±1.3	107.7±1	2882.6±25.1	3135.4	75684.4±198.8	89322.7±1202.4
14	234.6±16.4	10091.2±173.4	10813.7±1251	862.5±16.6	22002	3960.3±397.9	552.9±23	6763.9±169.5	11277.1	81.5±2.2	61.6±2	1848.9±40.7	1992	75059.1±225.9	79161.7±409.2
15	401±32.8	5666.3±276.1	12978.2±1701.1	998.4±42.7	20043.9	2134±328.1	148.8±9.8	3527.9±195.1	5810.7	36.9±2.9	24.1±1.8	945.4±37	1006.4	76488.7±280.5	72269.4±308.9
16	329.1±23.5	4907.6±67.5	10852.4±1084.4	578.4±8.5	16667.5	4120±422.2	389.4±16.1	5704.9±110.1	10214.3	133.9±2.3	31.6±0.8	1034.5±17.8	1200	74625.9±71.3	73377.9±630.4
17	295.3±27	9560.5±328.1	12781.7±1782.6	1561.7±139	24199.2	5888.5±738.1	251.7±13.6	6547.7±305.3	12687.9	108.2±2	20.9±0.4	805.2±7.6	934.3	76721.2±93.8	86600.1±284
18	268.9±26.6	6586.4±90	10251±1217.6	480.2±7.1	17586.5	14861.7±198.7	283.2±7.9	9307.3±168	24452.2	157.6±3	35.4±0.5	1127±8.9	1320	79816.2±50.1	82262.4±330.7
19	457.4±42.6	5459.4±81	8174.7±1261.9	617.3±39.2	14708.8	1478±331.7	1018.2±53.8	5955.3±186.7	8451.5	76.9±1.5	7.1±0.6	508.6±8.1	592.6	79354.3±82	80762.3±361.8
20	198.6±10.7	1398.2±66.2	6359.3±646.9	629±22.8	8585.1	8856.4±261	218.7±13.5	1216.1±63.5	10291.2	ND	ND	69.6±1.1	69.6	59930.6±133.3	50225.6±431.4
21	90.3±5.0	2021.9±19.3	3025.3±133.2	251.8±3.5	5389.3	979.6±136.6	115.2±1.5	1766.4±17.6	2861.2	ND	ND	67.2±1.2	67.2	52880.4±176.2	41268.3±158
22	152.9±12.5	7340.8±212.4	7127.5±635	892.7±57.8	15513.9	1533.8±47.1	223.7±4.5	4260.3±149.9	6017.8	48.1±1.0	ND	391.8±6.6	439.9	73956.8±163.1	69165.4±273.8
23	186.4±11.6	1632.4±21.2	5294.6±368.6	192.1±15.1	7305.5	2904.6±323.2	83.5±1.8	4550.8±35.9	7538.9	ND	ND	86.8±1.8	86.8	66699.4±255.7	50705.6±137.9
24	425.7±56.8	2037.6±114.1	9670.9±1206.8	652.8±65	12787	1808.1±87.4	1008.2±68.7	4707.8±242.8	7524.1	ND	ND	142±3.8	142	72908.9±163.7	63678.3±89.9
25	399.6±50.8	4129±120	12342.6±1556.8	723.1±62.7	17594.3	1717±91.1	112.5±0.9	1186.1±33.2	3015.6	ND	ND	49.6±1.1	49.6	75823±33.6	64700.5±345.8
26	349.4±49.4	2987.7±34.8	8558.5±1305.1	348.4±4.7	12244	1400.9±86.8	94.1±3.5	948.1±35.9	2443.1	ND	ND	37.6±0.9	37.6	69458.7±251.2	51678.7±227.5
27	202.2±33.9	11149.7±311.2	10624.4±1796.5	1689.4±75	23665.7	1380.5±79	176.4±9.9	4641.7±153.2	6198.6	295.2±9.3	54.5±2	1418.3±41.1	1768	76942.2±71.1	68499.9±854.7
28	611.7±98.5	4973.9±197.9	19957.2±2783.7	2217.8±89.5	27760.6	4015±199.5	767.2±40.9	7241.4±264.3	12023.6	126.5±1.7	21.4±0.5	815.7±9.5	963.6	79708.1±266.9	97028.5±158.1
29	187.7±31.7	1428.6±49.6	4904.6±872.5	426.4±11.7	6947.3	2333.3±102.3	33.5±0.5	1447.2±37.7	3814	ND	ND	21.3±1.2	21.3	51911.9±120.9	41480±72.2
30	376.2±51.7	1633.9±89.9	9344.2±1387.1	594.8±45.3	11949.1	885.3±96.8	53.4±4.7	984.4±75.2	1923.1	ND	ND	201.8±7.5	201.8	67399.7±263	49449.8±146.4
31	374.7±59.5	6776.3±241.6	15118.4±2587.3	1164.6±100.3	23434	2157.6±163	131.6±8.1	4488.4±197.4	6777.6	180.8±4.5	37.5±1.2	1139.8±17.5	1358.1	78912.5±90.5	83348.8±313.7
32	136.3±19.6	3093.6±22.3	5262.8±777.3	749.1±31.1	9241.8	1012.9±37.5	138.3±7.4	3124.5±34.7	4275.7	42.4±2.3	ND	345.8±9.5	388.2	68973.5±204.2	48994.8±287.3
33	262.3±30	4293.2±164.5	8261.9±1245.8	947.2±48.3	13764.6	960.3±70.6	107.4±8.2	3026.4±157.4	4094.1	45.6±0.9	ND	356.6±2.5	402.2	69376.1±171.9	60669.3±546.6
34	569.2±71.3	2208.4±86.1	10979.5±1699.4	350.7±32.5	14107.8	5692.4±290.1	233.5±11.1	3078.8±115.4	9004.7	18.7±1.7	ND	228.8±3	247.5	71588.1±99.1	65895.4±988.5
35	164.4±23	2449.7±31.8	5473.9±740.4	946.2±7	9034.2	2584.1±135.2	91±3.6	2715±44.2	5390.1	ND	ND	130.8±3.7	130.8	63579.6±195.7	45799.9±197.3
36	144.9±11.3	1706.4±13.3	4089.3±430	203.5±2.8	6144.1	2991.4±212.9	134.2±3	4704.1±84.5	7829.7	ND	ND	115.3±1.1	115.3	62816.7±659	39528.5±110.2
37	166.1±26.9	4001.5±225.7	5305.1±1071.1	748.5±41.4	10221.2	2021.4±96.4	63±4.4	2253.4±130.5	4337.8	ND	ND	172.9±3.5	172.9	67298.8±156.3	52515.3±250
38	146.1±21.7	1731.9±51.3	3861.9±639.8	272.3±7.7	6012.2	1955±86.3	205.3±12.4	2139.6±66.1	4299.9	ND	ND	47.4±1.5	47.4	55474.9±122.2	34162.9±144.2
39	157.6±27.5	5510.4±124.2	6884.2±1174	790.4±11.3	13342.6	5337.6±289.1	212.7±6	6129.8±143.4	11680.1	52±4.3	ND	350.3±17.4	402.3	70975.1±83	63176.3±342.6
40	97.1±16.0	3467.1±82.1	3277.3±626.9	447.1±11.3	7288.6	1735.3±166.5	79±3.4	3388.4±86.3	5202.7	32.8±5.2	ND	249.4±6.0	282.2	61110.7±250.9	47243.2±176.9
41	219.1±33.3	9129.3±422.7	10950.8±2169.5	1015.5±71.4	21314.7	7425.5±207.7	166.8±11.7	6611.3±339.3	14203.6	161.2±6.3	21.6±1.8	809.2±30.2	992	79185.2±131	81470.8±445.9
42	331.6±56.9	9984.3±486.9	13100.8±2481.1	1214.2±163.2	24630.9	5731±92.8	145.6±8.7	5014.4±264	10891	102.8±2.2	8.1±0.6	552.0±11.2	662.9	79654±126.2	84894.1±276.4

43	53.5±13.1	37.6±10.9	534.2±131.6	20.5±1	645.8	111.5±25.7	19.1±1.6	ND	130.6	ND	ND	ND	ND	12656.8±20.2	20642.5±112.7
44	52.9±8.3	162.9±12.1	574.2±120.7	33.8±0.7	823.8	325.3±17.7	33.6±1.9	168.4±13.4	527.3	ND	ND	ND	ND	15130.9±112.9	25685.6±129.4
45	59.9±13.1	101±14.1	632.1±156.5	38.6±6.7	831.6	275.5±22.5	32.1±2.7	117.6±15.5	425.2	ND	ND	ND	ND	14716.7±52.1	25954.4±90
46	442.8±92.8	3943.4±184.5	14050.2±934.5	317.2±17.7	18753.6	7234.7±976.4	709.3±50.1	8179±516.5	16123	209.5±7.2	21.6±0.8	839.9±13.2	1071	80040.9±136.8	73408.2±91.3
47	40.6±5.1	209.8±20.7	530.6±114.7	58.3±3.7	839.3	261±94.9	393.9±38.9	577.6±43.7	1232.5	ND	ND	51.8±3.7	51.8	23456.7±43	28161.8±187.4
48	70.2±11.7	251.1±25.9	787.4±35.7	71.1±5.2	1179.8	ND	373.5±33.3	410.5±28.5	784	ND	ND	ND	ND	23729.8±43.3	37344.5±237.6
49	25.6±5.6	31.8±0.9	337.1±22.9	22.6±1.9	417.1	207.9±35.4	239.7±21	99.3±16.6	546.9	ND	ND	ND	ND	15256.6±58.3	33900.8±144.5
50	37.4±2.7	153.7±1.8	421.4±73.7	27.4±0.9	639.9	106.6±17.7	113.4±4	110±3.7	330	ND	ND	ND	ND	14895.1±85.9	28645.3±235.5
51	71.8±12.1	156.6±16.6	719.1±159.1	39±2	986.5	116.3±33.8	51.5±3.2	ND	167.8	ND	ND	ND	ND	16284.6±24.2	28669.8±43.7
52	27±5.7	ND±6.2	360.1±89.2	16.2±0.4	403.3	108.2±28.9	124.2±13.7	ND	232.4	ND	ND	ND	ND	13008.2±49.6	24928.7±113.6
53	71.4±10.7	168.7±17.2	772.6±129.2	41.7±2.6	1054.4	160.8±25.2	95.9±7.9	128.8±11.5	385.5	ND	ND	ND	ND	18435±37.4	29491.2±193.2
54	279.9±44	9854.6±314.6	7853.8±1433.7	697.7±21.2	18686	7928.2±622.6	853±28.9	7941.5±207	16722.7	329.1±1.4	ND	1062.5±4.5	1391.6	80351.2±124.4	81901.5±360.7
55	37.0±8.2	ND	399.9±76.5	ND	436.9	574.5±67.5	48.3±3.3	ND	622.8	ND	ND	ND	ND	15189.1±162.9	28787.6±48.6
56	38.9±4.6	253.7±11	564.1±106	41.8±1.6	898.5	346.2±19.3	55.9±2.8	221.6±15.2	623.7	ND	ND	ND	ND	14543.9±55.8	28242.6±71.6
57	125.1±17.8	490.7±21.6	1362.6±220.3	46.9±3.6	2025.3	127.5±25.7	243.4±14.1	270.1±12.8	641	ND	ND	ND	ND	28815.5±80.2	37264±107
58	54.2±7.9	102.2±9.4	639.9±122.3	43.4±0.2	839.7	455.5±16.1	43.3±2.8	233.6±24.6	732.4	ND	ND	ND	ND	14467±50.8	27313.3±34
59	107±18.9	314.4±27.5	1020.5±175.1	80.1±5.3	1522	267.4±56.3	505±49.5	1178.2±50.5	1950.6	ND	ND	134.2±2.8	134.2	32642.1±36.2	35807.3±221.9
60	67.7±9.6	265.6±24.3	712.8±114.8	49.3±4.1	1095.4	152.2±36.4	211.8±15.6	282±21.7	646	ND	ND	6.3±0.5	6.3	19729.6±117.8	31066±93.2
61	48.8±7.7	82.4±11.1	550.2±81.7	32.7±3.4	714.1	153.3±25.7	276.6±14.7	208.1±15.5	638	ND	ND	ND	ND	15013.5±59.2	33708±51.7
62	57±12.3	235±21.5	634.8±129	39.2±2.8	966	114.7±35.4	74.3±6.4	129.4±4.7	318.4	ND	ND	ND	ND	14250.1±47.1	23025±73.2
63	41.8±10.1	197.8±30.5	639.5±128.1	59.7±12	938.8	220.8±77.2	124.2±11.4	184.4±28.8	529.4	ND	ND	ND	ND	14459.4±26.1	23002.7±108.5
64	213.8±36.6	4886.7±293.4	8805.5±1522.5	640.8±31.5	14546.8	4068.1±583.6	725±39.3	7576.3±372.2	12369.4	148.7±3.5	33.7±0.8	993.1±80.9	1175.5	79354.2±109.7	64442.9±323.9
65	236.5±41.2	469.5±16	1344.1±227.6	71.8±4.6	2121.9	254±48.4	1126.3±49.4	812.5±32.6	2192.8	ND	ND	ND	ND	42845.9±205.4	41012.9±150.4
66	56.6±6.9	178.2±12.1	635.1±123.3	35.6±1.6	905.5	212.7±62.7	127.6±4.1	274.2±14.4	614.5	ND	ND	19.1±0.5	19.1	15515±118.4	25506.6±106.6
67	41.1±14.8	433.6±30.3	737.4±149.6	67.7±5.1	1279.8	1288.3±247.6	225.8±19.6	987±78.1	2501.1	ND	ND	ND	ND	24640.5±213.5	28677.9±64.3
68	36.8±15.1	87.1±14.1	542.3±114.9	24.1±1.3	690.3	301.2±77.2	69.3±1.9	193.5±24.9	564	ND	ND	ND	ND	13318.2±84.9	24247.6±149.6
69	50.4±16.7	378.4±38.3	779.5±141.2	73.5±5.3	1281.8	754.5±140.5	189.7±16.8	721.7±66.9	1665.9	ND	ND	9.4±1.2	9.4	23687.7±73.4	29763.8±51.1
70	85.5±18.5	20.8±3.5	685.8±142.8	32.1±2	824.2	688.6±27.3	76.7±4.9	162.4±16.8	927.7	ND	ND	ND	ND	14525.1±239.4	23957.7±105
71	40.5±7.9	100.2±7.9	512.9±86.6	25.2±0.9	678.8	ND±	113.8±1.3	130.9±14.1	244.7	ND	ND	ND	ND	14194.3±80.1	22674.1±16.8
72	38.3±15.6	114.8±14.4	512.8±123.7	43±3.2	708.9	331.7±46.6	129.8±14.5	212.9±18.1	674.4	ND	ND	ND	ND	12850.7±50	23507.5±55.2
73	47.6±10.6	83.9±13.8	529.6±136	23.5±1.4	684.6	256.2±28.9	33.2±2.8	110.5±16.2	399.9	ND	ND	ND	ND	13290±75.6	27225.8±66.4
74	64.6±0.8	421.6±30.8	729.3±160.1	86.7±7.7	1302.2	359.1±32.2	86.8±3.7	550.4±48	996.3	ND	ND	4.7±0.7	4.7	23268.6±194.7	28972.7±82.3
75	49.3±11.5	125.3±17.9	577.9±134.4	28.2±2.4	780.7	386.4±53.1	69.4±8.2	139.1±21.7	594.9	ND	ND	ND	ND	13654.5±179.8	26588.1±46.1
76	50.5±19	897.7±79.7	892.7±197.7	160.6±5.1	2001.5	145.8±42.2	247.5±25.5	649.1±47.8	1042.4	ND	ND	71.9±6.7	71.9	31135.7±273.5	31077.7±264.2
77	42.2±10.3	173.2±35.9	509.3±99.9	35.8±4.9	760.5	99.7±18.4	45.2±5.4	45.4±18.3	190.3	ND	ND	ND	ND	14230.3±91.1	29033.8±17.2
78	71.6±15.3	145.3±32.2	736.2±148.2	28±2.6	981.1	94.3±16.7	83.5±8.4	102.6±19	280.4	ND	ND	ND	ND	16641.8±56.4	32777±117.2
79	64.7±12.7	147.6±22.6	782.1±132.3	33.9±3.2	1028.3	114.7±9.5	252.2±27.3	494.4±38.9	861.3	ND	ND	31.5±1.6	31.5	17538.8±60	33453.6±327.7
80	57.2±12.3	327.5±33.2	702.8±137.5	48.8±4.7	1136.3	395.5±61.3	179.3±17.1	674.1±49.6	1248.9	ND	ND	73.3±4.5	73.3	22643.8±57	31940.5±95.5
81	34.4±3.6	115.3±2.3	491±4.3	24.2±0.3	664.9	468.4±6.4	79.6±1	130.9±0.5	678.9	ND	ND	ND	ND	14187.6±48.6	24444.6±64.8
82	26.2±4.4	789.4±12.1	564.1±15.7	155.1±2.1	1534.8	79.3±3.2	212.4±1.4	526.2±12.3	817.9	ND	ND	59±3.6	59	27383.6±169.6	27438.1±52.2
83	40.6±2	265.2±0.4	514.6±9.9	37.4±0.6	857.8	421.8±8	31.2±0.4	170.8±2.1	623.8	ND	ND	ND	ND	17458.8±100.3	25713.2±313.7
84	263.7±46.5	4215.3±112.9	8355.2±1981.4	1006±104.1	13840.2	4384.8±273.3	910.2±41.7	5897±214.2	11192	ND	ND	581.6±23.8	581.6	70220.3±59.5	63307.5±120.5
85	104.1±24.3	359.7±16.2	1811±409.5	165.1±0.5	2439.9	ND±	89.7±4.9	460.7±27.5	550.4	ND	ND	12.4±0.4	12.4	23370.4±36.7	25479.6±59.8
86	227.6±60.9	14937.1±217.3	13693.9±2736.2	830±27.1	29688.6	6190.8±1214.5	569.5±15.9	11453.4±419.2	18213.7	133.8±2.5	107.8±4.6	3986.2±99.5	4227.8	80349.7±130.8	105062.8±426.1
87	107±17.1	2380.7±48.2	3598.8±645.5	496.4±18.6	6582.9	922.8±132	229.2±8.9	2229±66.2	3381	ND	ND	253.2±4.9	253.2	54472.5±162	41433±393
88	282.1±48.7	7929.9±109.6	11683.6±2232.3	1497.6±47.9	21393.2	2038.6±277.3	367.3±13.3	5419.2±120.8	7825.1	41.8±1.1	38.1±0.7	1530.4±20.6	1610.3	74041.5±64	79562.4±407.2
89	131.6±20.5	3261.8±149.5	3530.3±592.5	439.1±22.2	7362.8	1379.4±153.7	371.6±17.7	2948±71.7	4699	ND	ND	432.7±3.7	432.7	61821.5±112.2	45358±367.8

90	294.9±48	4688.7±102.9	9833.7±1552.5	436.8±24.5	15254.1	5429.8±1014.5	916.8±38.4	13264.8±384.5	19611.4	70.8±3.5	51.8±1.4	2043.9±29.9	2166.5	71232.8±337.2	72287.4±289.9
91	126.8±21.5	3837.4±137.4	4574.8±1029.5	390.1±13.3	8929.1	5687±764.9	308.9±15.1	5383.9±225.6	11379.8	ND	ND	431.6±12.6	431.6	65613.6±229.7	45977.1±109
92	169.4±24.3	7143.3±165.6	7485.5±1185.8	852.8±40.8	15651	5915.3±1592.4	1841.8±197.4	14343.4±485.9	22100.5	85.4±1.6	51.1	1999.8±14.5	2136.3	78438.2±129.1	76222.7±301.4
93	130.3±23.1	4426.7±104.1	5314.2±1031	419.4±13.9	10290.6	2172.6±459.1	146±23.4	4055±252.9	6373.6	ND	ND±0.3	545.3±5.4	545.3	73953.3±62	55883±113
94	149.1±29.1	4033.5±661.1	4746±889.5	634.9±53	9563.5	1679±255.6	340.5±1.3	3449.3±63.4	5468.8	ND	ND±0.2	481.4±10.1	481.4	68100.5±127.8	54043.3±161.2
95	121±17.9	408.4±19.7	1853.3±304.5	101.9±8	2484.6	798.5±125.3	180.6±7.6	722.2±32.7	1701.3	ND	ND	11.1±0.1	11.1	33427.2±28.9	32919.8±45.6
96	210.3±41.7	610.7±48.5	2984.6±507.3	139.8±7.6	3945.4	2116.8±66	166.8±13.1	790.5±52.2	3074.1	ND	ND	ND	ND	38423.8±61.3	31491.7±58.8
97	319.8±58.2	6994.5±36.4	11183.5±2057.4	626±15.1	19123.8	5120±673.9	665.3±28	10844.9±399.5	16630.2	113±2.3	57.1±1.2	1924.4±17.5	2094.5	80370.7±64.1	74886.9±233.9
98	427.8±70.2	7144.6±75.5	13069.7±2167.4	447.7±16.5	21089.8	9390.1±830.1	489.2±14.8	8985.9±216	18865.2	80.5±2.3	35.9±0.5	1397.7±17	1514.1	79648.2±161.2	68773.9±190.3
99	153.8±20	3111.7±59.2	4398.7±777	678.4±20	8342.6	948.5±150.6	258.6±8.4	2700.9±32.2	3908	ND	ND	434.1±7.5	434.1	62998.4±169.5	40880.1±222.5
100	99.6±12.5	3922.2±82.3	3350.4±556.8	736.9±15.5	8109.1	881±200.1	321.5±10.7	3161.5±84.2	4364	25.1±1	4.6±0.6	566.8±10.2	596.5	65334.6±111.7	46852.5±195.6
101	282.3±40.3	3297.2±9.2	8686.8±1116.2	308.6±24.5	12574.9	3732±856.5	620.7±44.6	9973.9±207.8	14326.6	80.9±4.4	32.8±1	1278.6±28.8	1392.3	72649.2±172.8	61530.2±373.1
102	191.2±27.2	10274.2±143	9044.9±1596.7	1353.6±54.4	20863.9	2811.4±468.5	485.7±16.1	9905±228.1	13202.1	422.9±6.9	201.3±2.2	5013.6±14.4	5637.8	81310.3±211.1	90737.5±230.6
103	185.6±27.6	7435.7±74.5	8719.3±1466	1053.5±68.6	17394.1	6448±714.9	447.2±16.3	7780.1±161.8	14675.3	47.4±1.7	11.9±1.3	751.1±14.9	810.4	75552.7±133.7	70445.4±335.7
104	176±23.9	7396.8±137.3	8889.3±1458.2	826.7±11	17288.8	9173.6±1097.7	522.4±15.7	9775.8±194.1	19471.8	69.9±2.1	19.8±1.7	944.4±23.7	1034.1	74776.3±107.8	64867±644.7
105	466.1±66	4019.8±110.3	14448.3±1885	459.9±17.2	19394.1	12142.5±2559.3	1280.1±116.3	22148.5±702	35571.1	338.9±4	140.8±4.4	3759.2±71.4	4238.9	80381.5±71.4	77323.1±238.9
106	86.8±15.3	2388.2±60.8	2627.5±386.5	366.2±9.9	5468.7	2121.1±74.9	289.2±10.6	3987.4±90.5	6397.7	ND	ND	140.8±3.0	140.8	42517±70.8	30658.8±61.3
107	485.2±60.9	5267.6±63.5	10573.3±1639.7	1054.9±52.9	17381	3019.3±80.7	244.3±8.9	7208.3±105.3	10471.9	ND	ND	162.3±1.7	162.3	59378±605.2	42327.1±472.1
108	278.1±44.5	14676.3±81.3	14187.6±2073.8	2209.3±114	31351.3	7429.8±1238.5	2691.6±138.7	31121±395.2	41242.4	32.7±1.4	52.0±0.5	2721.5±10.1	2806.2	79116.5±91.2	79292.8±574.9
109	109.2±2.1	2775.8±21.8	2235.9±4.5	749.9±21.5	5870.8	585.7±4.8	210.2±2.8	2010.4±2.4	2806.3	ND	ND	33.6±3.7	33.6	27719.9±45.1	31181±11.2
110	113.7±14.9	4006.1±33.4	3888.7±550.7	478.7±5.1	8487.2	5177.9±99.5	513.2±7.6	6395.1±118.2	12086.2	ND	ND	40.0±1.5	40.0	45623.5±48.2	36073.8±191.1
111	46.4±1.6	2007.5±9.5	1283.9±19.8	286.8±0.7	3624.6	669.3±168.8	392.5±17.1	3745.6±5	4807.4	ND	ND	245.3±7.5	245.3	49440.2±26	34598.8±113.4
112	412.7±56.1	1777.4±50.4	7146.8±1087.7	703±62.6	10039.9	10449.1±384.3	320.4±13.2	2086.7±76.8	12856.2	14.2±1.2	16.5±0.6	1135.4±7.8	1166.1	73836.5±52.8	57335.7±245.5
113	78.9±11.1	2764.4±12.2	2698.5±297	448.4±1.9	5990.2	4364.4±287.1	342.6±18.6	3435.2±31.9	8142.2	ND	ND	164.6±0.9	164.6	64080.5±37	43672.3±173.8

ND = Not detected, values are either below the limit of quantification or limit of detection. 3-CQA, 3-*O*-caffeoylquinic acid; 5-CQA, 5-*O*-caffeoylquinic acid; 2,3-DCTA, 2,3-di-*O*-caffeoyltartaric acid; 3,5-DCQA, 3,5-di-*O*-caffeoylquinic acid; Q3-G, quercetin 3-*O*-glucuronide; L7-G, luteolin 7-O-glucuronide; Q3-6''MG, quercetin 3-*O*-(6''-*O*-malonyl)glucoside; C3-G, cyanidin 3-*O*-glucoside; C3-3''MG; cyanidin 3-*O*-(3''-*O*-malonyl)glucoside; C3-6''MG, cyanidin 3-*O*-(6''-*O*-malonyl)glucoside; ABTS, 2,2′-azinobis-(3-ethylbenzothiazoline-6-sulfonic acid) radical scavenging activity; TPC, total phenolic content.
